# Extensive Use of RNA-Binding Proteins in *Drosophila* Sensory Neuron Dendrite Morphogenesis

**DOI:** 10.1534/g3.113.009795

**Published:** 2013-12-17

**Authors:** Eugenia C. Olesnicky, Darrell J. Killian, Evelyn Garcia, Mary C. Morton, Alan R. Rathjen, Ismail E. Sola, Elizabeth R. Gavis

**Affiliations:** * Department of Molecular Biology, Princeton University, Princeton, New Jersey 08544; † Department of Biology, University of Colorado at Colorado Springs, Colorado Springs, Colorado 80918; ‡ Biology Department, The Colorado College, Colorado Springs, Colorado 80903

**Keywords:** dendrite morphogenesis, dendritic arborization neuron, Drosophila, RNA-binding proteins, RNAi screen, post-transcriptional regulation

## Abstract

The large number of RNA-binding proteins and translation factors encoded in the *Drosophila* and other metazoan genomes predicts widespread use of post-transcriptional regulation in cellular and developmental processes. Previous studies identified roles for several RNA-binding proteins in dendrite branching morphogenesis of *Drosophila* larval sensory neurons. To determine the larger contribution of post-transcriptional gene regulation to neuronal morphogenesis, we conducted an RNA interference screen to identify additional *Drosophila* proteins annotated as either RNA-binding proteins or translation factors that function in producing the complex dendritic trees of larval class IV dendritic arborization neurons. We identified 88 genes encoding such proteins whose knockdown resulted in aberrant dendritic morphology, including alterations in dendritic branch number, branch length, field size, and patterning of the dendritic tree. In particular, splicing and translation initiation factors were associated with distinct and characteristic phenotypes, suggesting that different morphogenetic events are best controlled at specific steps in post-transcriptional messenger RNA metabolism. Many of the factors identified in the screen have been implicated in controlling the subcellular distributions and translation of maternal messenger RNAs; thus, common post-transcriptional regulatory strategies may be used in neurogenesis and in the generation of asymmetry in the female germline and embryo.

Dendrites are neuronal structures that receive sensory and synaptic input, allowing a neuron to perceive and respond to its surrounding environment. Dendrites display diverse and often complex arborization patterns that are integral to neuron function, as they determine the type of sensory or synaptic input a neuron is capable of processing. Although a myriad of dendritic arbor morphologies has been reported, the molecular mechanisms that regulate dendrite morphogenesis and the establishment of specific arborization patterns are only partly understood. Recent studies have begun to identify the molecular components that are involved in generating cell type specific arborization patterns. These studies have largely focused on the roles of transcription factors that regulate the overall arborization architecture of specific neural subtypes (Parrish *et al.* 2006; Ou *et al.* 2008; Jan and Jan 2010; Iyer *et al.* 2013). Because dendritic compartments often are distant from the nucleus, transcriptional control may be far upstream of the local events that regulate outgrowth and branching. Thus, studies focusing on understanding the local mechanisms necessary to direct branching and outgrowth are needed to shed light on mechanisms governing dendrite elaboration.

Cellular machinery dedicated to regulating mRNA processing, localization and translation is often employed in the generation of developmental asymmetry as during the establishment of embryonic body axes and specification of the germline (Martin and Ephrussi 2009; Richter and Lasko 2011). The development and function of polarized cells like neurons may rely on similar mechanisms, including the transport and localization of translationally silent mRNAs and local translational activation of mRNAs at the target destination. A large number of mRNAs, as well as a full complement of translational machinery, have been shown to reside in the dendrites of differentiated neurons (Martin and Zukin 2006). Moreover, local protein synthesis within dendrites is required for activity dependent synaptic refinement and strengthening (Richter and Klann 2009; Doyle and Kiebler 2011). Less is known about the role of post-transcriptional gene regulation in dendrite morphogenesis and in the establishment and maintenance of complete and functional neural circuits. However, recent studies of dendritic arborization (da) neurons in the *Drosophila* peripheral nervous system have shown that translational regulators including Nanos (Nos), Pumilio (Pum), and the Fragile X mental retardation protein (FMRP) direct some aspects of dendrite development and branching (Ye *et al.* 2004; Brechbiel and Gavis 2008; Bianco *et al.* 2010; Auerbach *et al.* 2011; Olesnicky *et al.* 2012).

Inappropriate mRNA regulation and altered rates of protein synthesis have also been described in neurological disorders where dendritic morphology or density is affected. Lesions that silence the *fmr1* gene, which encodes FMRP, cause Fragile X syndrome and are linked to autism spectrum disorder (Auerbach *et al.* 2011) and mice lacking FMRP show increased levels of protein synthesis and supernumerary dendritic spines (Bagni and Greenough 2005). Mutations in the tuberous sclerosis complex genes *Tsc1* and *Tsc2*, which indirectly function to activate mRNA translation, are also associated with autism spectrum disorder and other intellectual disabilities. In contrast to *fmr1* knockout mice, *Tsc2+/−* mice exhibit decreased dendritic spine numbers and decreased protein synthesis (Orlova and Crino 2010; Auerbach *et al.* 2011; Santoro *et al.* 2012). These findings suggest that protein synthesis must be tightly regulated for the development of dendritic structures and for the preservation of normal synaptic function. Therefore, a thorough understanding of the contribution of post-transcriptional gene regulation to dendritic morphogenesis will aid in the development of therapeutic agents for the treatment of neurological disorders.

*Drosophila* class IV da neurons, which function in nociception and light avoidance (Hwang *et al.* 2007; Xiang *et al.* 2010), have characteristic complex dendritic arbors that cover the majority of the larval epidermis in a non-overlapping manner termed tiling (Grueber *et al.* 2002). To better understand the role of mRNA regulatory mechanisms in dendrite morphogenesis, we investigated the functions of genes encoding RNA binding proteins (RBPs) and translational regulators in the elaboration of the dendritic branching patterns of class IV da neurons by performing a cell type-specific RNA interference (RNAi) screen. Here we present the overall results of the screen and focus in depth on the phenotypes resulting from knockdown of 23 RBPs. We find that RBPs and translation factors contribute broadly to dendrite morphogenesis and to the development of complex arborization patterns. Several categories of RBPs, most notably splicing and translational initiation factors, were associated with distinct and characteristic phenotypes, suggesting that different morphogenetic events are best controlled at particular steps in post-transcriptional mRNA metabolism. Additionally, many of the factors identified in the screen have been previously implicated in controlling the subcellular distributions and translation of maternal mRNAs in the oocyte and early embryo. Common post-transcriptional regulatory strategies may thus be employed in neurogenesis and in the generation of asymmetry in the female germline and embryo.

## Materials and Methods

### Fly strains and genetics

Virgin females from the *GAL4^477^*, *UAS-mCD8:GFP* (Grueber *et al.* 2003) and *ppk-GAL4*, *UAS-mCD8:GFP* (Grueber *et al.* 2007) strains were crossed to *UAS-RNAi* males to generate larvae expressing RNAi hairpins together with the mCD8-GFP membrane marker in class IV da neurons. For each GAL4 driver, control larvae expressing the mCD8-GFP membrane marker alone were generated by outcrossing to wild type. Individual *UAS-RNAi* strains obtained from the Vienna *Drosophila* RNAi Center (VDRC; Dietzl *et al.* 2007), the Transgenic RNAi Project (TRiP; Harvard Medical School), and National Institute of Genetics (NIG; Japan) stock centers are listed in Supporting Information, Table S1. The *UAS-nosRNAi* line is described in Menon *et al.* (2009). For experiments using *GAL^477^*, animals were maintained at 25°. Experiments using *ppk-GAL4* were performed at 29° to increase GAL4 efficiency. *UAS-Dcr2* was not used to enhance RNAi because it produced dendrite defects on its own.

### Imaging and quantification of dendrite morphology

Dendrite morphology was examined in wandering larval stages, corresponding to approximately 108−120 hr after egg laying. Larval fillet preparations were fixed as described (Ye *et al.* 2004) using electron microscopy grade formaldehyde (Polysciences) and immunostained with 1:350 Alexa Fluor 488 rabbit anti-GFP (Invitrogen), mounted in 70% glycerol, and imaged on either a Leica SPE confocal microscope or a Leica SP5 confocal microscope using a 40x/1.25 NA oil objective. ddaC neurons from the second through fifth abdominal segment were imaged and scored. All lines were scored blindly during the screening process, with at least eight neurons, each from a different larva, evaluated qualitatively per line.

The number of branch points and the total branch length were quantified in Z series projections of ddaC neurons imaged on a Leica SP5 confocal microscope. Images shown in Figure 2, Figure 3, Figure 4, and Figure 5 (top row) were acquired using the SP5. For each RNAi line, six to nine neurons were quantified, each from a different larva. A single author manually performed all branch point quantifications from the original images. Branch length was quantified from neuronal tracings using NeuronJ (Meijering *et al.* 2004). Statistical significance was determined by performing the Student’s *t*-test. For wild-type ddaC neurons visualized with mCD8-GFP expressed using *ppk-GAL4* (control neurons), approximately 400 branch points were routinely detected within the field of view.

## Results

### Class IV da neuron screen for RNA regulatory proteins that function in dendrite *morphogenesis*

The *Drosophila* genome encodes at least 400 proteins annotated as mRNA binding proteins, proteins with known RNA binding domains, and/or translation factors excluding ribosomal proteins (Gamberi *et al.* 2006; this study). Transgenic strains that express RNAi constructs under control of an upstream activating sequence (UAS) have been generated for the majority of these genes (Dietzl *et al.* 2007). Our screen encompassed 323 such *UAS-RNAi* lines representing 302 genes. Among these were four genes encoding RNA binding proteins that we had previously shown through mutational analysis to be required in class IV da neurons and thus served as positive controls: *nanos* (*nos*), *pumilio* (*pum*), *smaug* (*smg*), and *glorund* (*glo*) (Ye *et al.* 2004; Brechbiel and Gavis 2008; Olesnicky *et al.* 2012).

*UAS-RNAi* transgenes were expressed using *GAL4^477^* (Grueber *et al.* 2003), which drives expression in differentiated class IV da neurons late in embryogenesis and during larval development. Neuronal morphology was monitored using the mCD8-GFP marker, also under *UAS* control. Expressing *UAS-RNAi* transgenes post-differentiation favored the identification of genes required for dendrite morphogenesis rather than those involved in earlier stages of neurogenesis for cell fate specification and differentiation. Additionally, using cell type specific RNAi-mediated knockdown facilitated the analysis of genes that function cell autonomously within class IV da neurons but whose mutation causes lethality due to functions in other tissues as well.

*UAS-RNAi* lines for three of the four positive controls were successfully identified in the blind primary screen as causing a reduction in the number of dendrites; we did not detect a phenotype using two different *UAS-RNAi* transgenes targeting *pum*. An additional 122 lines also exhibited dendritic phenotypes and these were subsequently retested using a different class IV da neuron specific driver, *ppk-GAL4* (Grueber *et al.* 2007), to confirm the phenotypic results. In this secondary screen, *UAS-RNAi* lines targeting 88 RBP and translation factor encoding genes exhibited aberrant dendritic morphology, including alterations in the size of the dendritic field, the number of branches, branch length, and patterning of the dendritic tree (Table 1). Notably, all of these genes encode highly conserved proteins (Table S2). At the time the screen was performed, independent RNAi lines were only available for a small subset of the positive candidates. We were able to confirm dendrite defects for eight of them (Table 1). Moreover, we have validated two of the positive candidates, *brain tumor* (*brat*) and *4EHP*, with mutants and have shown that they function together with *nos* and *pum* to regulate dendrite morphogenesis (Olesnicky *et al.* 2012). Mutational analysis of two others, *oskar* (*osk*) and *rumpelstiltskin* (*rump*), also confirmed roles in class IV da neurons (Xu *et al.* 2013). While we have confirmed the RNAi phenotypes for a small number of genes using independent RNAi lines and mutants, it remains possible that some of the genes identified are false positives due to off-target effects of RNAi.

**Table 1:** Genes identified as positive

Gene	RNAi Stock Number
*alan shepard^a^*	VDRC 37863, NIG 32423R-2
*arrest^a^*	VDRC 107459
*bancal^b^*	VDRC 105271
*brain tumor^c^*	VDRC 105054, TRiP HMS01121
*CG3056^a^*	VDRC 101781
*CG4119^a^*	NIG 4119R-2
*CG4887^a^*	VDRC 105322, NIG 4887R-3
*CG5168^c^*	VDRC 110451
*CG5439^a^*	NIG 5439-R1
*CG5589^d^*	VDRC 108642
*CG5705^e^*	VDRC 108376
*CG5800^b^*	VDRC 103769
*CG6418^b^*	VDRC 108552
*CG6961^a^*	VDRC 109951, NIG 6961R-3
*CG7082^b^*	VDRC 103708
*CG7903^a^*	VDRC 106475, NIG 7903R-1
*CG9107^a^*	VDRC 109500
*CG10466^a^*	VDRC 104715
*CG10777^d^*	VDRC 109465
*CG11266^a^*	NIG 11266-R2
*CG11334^e^*	VDRC 109672
*CG11454^a^*	NIG 11454R-4
*CG11505^a^*	VDRC 105949, NIG 11505R-2
*CG11726^a^*	NIG 11726-R1
*CG12493^f^*	VDRC 102360
*CG14718^a^^,^^c^*	VDRC 105543
*CG14891^a^*	VDRC 102118
*CG18259^a^*	VDRC 50094
*CG32706^a^*	VDRC 109212
*CG34354^a^*	VDRC 102597
*CG40351^a^*	VDRC 40683
*cyclophilin-33^a^*	VDRC 108734
*Dbp73D^d^*	VDRC 108310
*Ddx1^d^*	VDRC 103365
*Dicer-1^a^*	VDRC 106041
*Dicer-2^d^^,^^f^*	TRiP 02636
*Disco Interacting Protein-1^f^*	VDRC 108186
*Drosha^f^*	VDRC 108026
*eEF1delta^e^*	VDRC 107007
*Ef1alpha48D^e^*	VDRC 104502
*Ef1alpha100E^e^*	VDRC 102736
*Ef1beta^e^*	VDRC 106636
*Efsec^e^*	VDRC 105437
*4EHP^e^*	VDRC 38399
*eIF-1A^e^*^,^*^g^*	VDRC 100611
*eIF-2alpha^e^*^,^*^g^*	VDRC 104562
*eIF-2beta^c^*^,^*^e^*	VDRC 105291
*eIF2B-delta^e^*	VDRC 104403
*eIF2B-epsilon^e^*	VDRC 34711
*eIF2B-gamma^e^*	VDRC 108083
*eIF3-S2* (*Trip1*)*^e^*	VDRC 103141
eIF3-S4 (CG8636, CG10881)*^a^*^,^*^c^*^,^*^e^*	VDRC 105325
*eIF3-S5^e^*	VDRC 101465
*eIF3-S8^e^*	VDRC 26664
*eIF3-S9^a^*	VDRC 107829
*eIF-3p40^e^*	VDRC 106189
*eIF-4A^d^*	VDRC 100310
*eIF4E-4^e^*	VDRC 107595
*embryonic lethal abnormal vision^a^*	VDRC 37915, TRiP JF03008
*eRF1^e^*	VDRC 45027
*found in neurons^a^*	VDRC 101508
*fusilli^a^*	VDRC 107575
*Gemin3^d^*	VDRC 49506
*glorund^a^*	VDRC 27752, NIG 6946R-1
*Gr98d^a^*	VDRC 106079
*helicase at 25E^d^*	VDRC 104481
*lethal(2)35Df^d^*	VDRC 108847
*loquacious^f^*	VDRC 108358
*mask^b^*	VDRC 103411
*mind bomb 2^c^*	VDRC 108947
*mtEF-Ts^e^*	VDRC 103791
*muscleblind^c^*	VDRC 105486
*nanos^c^*	Menon *et al.* (2009)
*oo18 RNA-binding protein*	VDRC 106257, NIG 10868R-1
*oskar*	VDRC 107546
*pitchoune^d^*	VDRC 106078
*rasputin^a^*	VDRC 109911
*Ribosomal protein S3^b^*^,^*^e^*	VDRC 10321
*rump^a^*	VDRC 44659
*SF2^a^*	VDRC 27776
*second mitotic wave missing^a^*^,^*^c^*	VDRC 105950
*smaug*	NIG 5263R-2
*smooth^a^*	VDRC 108351
*sans fille^a^*	VDRC 104334
*Spargel^a^*	VDRC 103355
*spoonbill* (*yu*)*^b^*	VDRC 105107
*squid^a^*	VDRC 32395
*Srp54^a^*	VDRC 51088
*U2 snRNP auxiliary factor 38*^c^	VDRC 110075
*x16^a^*^,^*^c^*	VDRC 100226

Positive candidates, including three positive control genes, are listed by their annotated gene name (FlyBase) or CG number where no name has been assigned. The protein eIF3-S4 is encoded by two identical genes. Thus, UAS-RNAi lines available for each one target the other. High throughput expression analysis (FlyAtlas Anatomical Expression Data, modENCODE Tissue Expression Data) shows CG8636 is ubiquitously expressed at high levels throughout development whereas CG10881 expression is largely restricted to testis, larval imaginal discs, fat body, and accessory gland. Thus, we suspect that the dendritic phenotype is due to knockdown of CG8638. Canonical RNA-binding motifs found in each protein are indicated as: ^*a*^ RNA recognition motif; ^*b*^ hnRNP K homology domain; *^c^* zinc finger; *^d^* DExH/D box; *^e^* translation factors; *^f^* double-stranded RNA-binding domain; *^g^* S1 RNAbinding domain. Translation factors are indicated by #. Homologs for genes listed are shown in Table S2.

### Phenotypic analysis of selected UAS-RNAi lines

Here we have selected 23 additional *UAS-RNAi* lines for more in depth phenotypic analyses. These lines were chosen because they produced more penetrant as well as characteristic phenotypes and target RBPs of diverse function or previously uncharacterized proteins. Overall dendrite length and the number of dendrite branch points, a measure of dendritic branching, were quantified from confocal Z-series projections of RNAi-expressing neurons and the results of this analysis are presented below. In addition to the number and length of branches, the patterning or distribution of dendritic arbors within the field can also determine the efficacy of gathering sensory information (Snider *et al.* 2010). Indeed, knockdown of a variety of RBPs produced defects in branch spacing or patterning of the dendritic tree in addition to defects in branch number and length. These phenotypes included clustering of dendrites into tufts, typically at the ends of main branches, and field coverage defects. The incomplete penetrance and range of patterning defects exhibited by each *UAS-RNAi* line, sometimes affecting only half of the dendritic tree, is likely due to the incomplete knockdown of gene function associated with RNAi. However, the large number of lines that showed neuronal patterning defects suggests that the establishment of arborization patterns requires the integration of a complex genetic network comprising many RBPs and translation factors.

#### Excessive branching:

**Figure 1:**
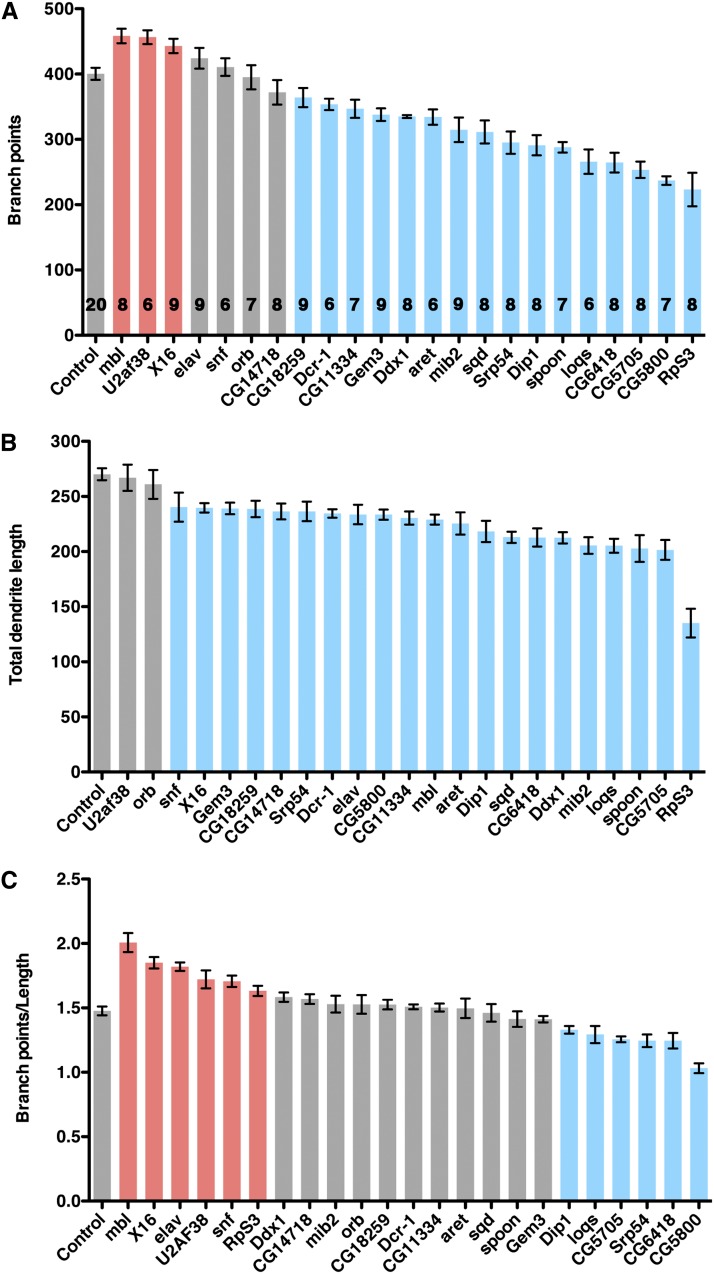
Quantification of dendritic defects. *ppk-GAL4* was used to express *UAS-mcd8:GFP* alone (control) or together with the indicated *UAS-RNAi*. (A-C) Graphs show mean values for branch point number (A), total dendrite length (B), and the ratio of branch points/dendrite length (C) for each *UAS-RNAi* line tested. The number of neurons analyzed for each RNAi line in (A-C) is indicated on the graph bars in (A). Error bars show the SEM. Data were analyzed using the Student’s *t*-test, and identical results were obtained using the Mann−Whitney *U* test. Bars are colored gray if the mean value is not significantly different from the control, red if the mean value is significantly greater than the control (*P* < 0.05), or blue if the mean value is significantly lower than the control (*P* < 0.05). RNAi, RNA interference; UAS, upstream activating sequence.

**Figure 2:**
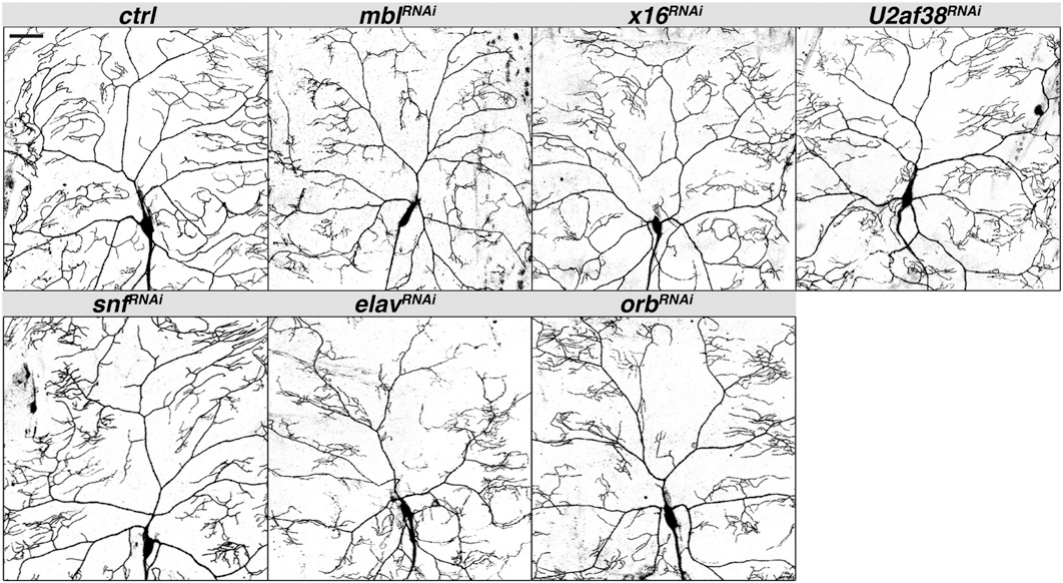
RNAi lines that produced an increase in branch number and/or density. Confocal Z-series projections of representative ddaC neurons with *ppk-GAL4* driving expression of *UAS-mcd8-GFP* alone (control) or together with the indicated *UAS-RNAi* transgene. Quantification of neurons of each genotype is shown in Figure 1. Although *orb^RNAi^* did not alter the number of branch points or total dendrite length, aberrant clustering of terminal branches produced local increases in branch density and gaps in coverage. Scale bar = 50 µm. RNAi, RNA interference; UAS, upstream activating sequence.

Knockdown of *muscleblind (mbl)*, *U2 small nuclear riboprotein auxiliary factor 38* (*U2af38*), and *x16* resulted in an increase in the number of branch points due to an excess of higher order branches (Figure 1 and Figure 2). Total dendrite length was either unchanged (*U2af38*) or decreased (*mbl*, *x16*), however, indicating that branches are shorter than in wild-type control neurons. The decrease in total dendrite length for *mbl* and *x16* reflects shortening of primary as well as higher order branches, such that the dendritic tree no longer covers the entire field. In contrast to the evenly spaced dendritic branches of control neurons, knockdown of all three genes also caused defects in the spacing of dendrites, with clustering of terminal branches and resulting gaps in coverage within the arbor.

Knockdown of two genes, *embryonic lethal abnormal vision* (*elav*) and *sans fille* (*snf*), had no affect on the number of branch points but did reduce total dendrite length, resulting in an elevated ratio of branch points to length like that of *mbl*, *U2af38*, and *x16* RNAi neurons. Similarly, the shortening and clustering of branches in *elav* and *snf* RNAi neurons produced coverage defects (Figure 1 and Figure 2).

#### Loss of branching:

**Figure 3:**
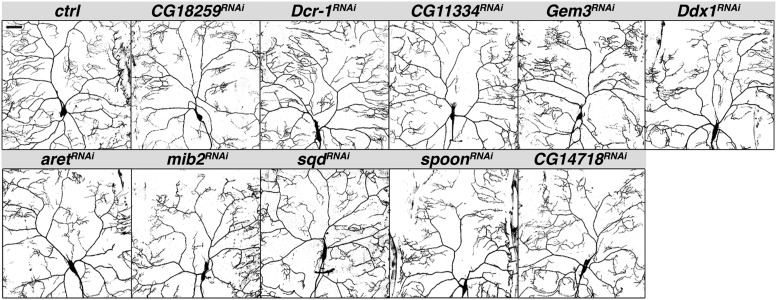
RNAi lines that produced branch loss and a concomitant decrease in dendritic length. Confocal Z-series projections of representative ddaC neurons with *ppk-GAL4* driving expression of *UAS-mcd8-GFP* alone (control) or together with the indicated *UAS-RNAi* transgene. Quantification of neurons of each genotype is shown in Figure 1. Note that *CG14718^RNAi^* did not affect the number of branch points but did reduce total dendrite length, producing coverage defects similar to those of the other RNAi lines shown. RNAi, RNA interference; UAS, upstream activating sequence.

Knockdown of *CG18259*, *Dicer-1* (*Dcr-1*), *CG11334*, *Gemin3* (*Gem3*), *Dead-box-1* (*Ddx1*), *arrest* (*aret*), *mind bomb 2* (*mib2*), *squid* (*sqd*), *spoonbill* (*spoon*; also known as *yu*) resulted in both branch loss and a concomitant decrease in total dendritic length (Figure 1 and Figure 3). With the exception of *CG11334*, *Gem3*, and *sqd* RNAi, primary branches were affected as well as higher order branches, such that the neurons did not cover the full receptive field. In the majority, loss of higher order branches and clustering of terminal branches also produced gaps within the tree. While knockdown of CG14718 did not reduce the number of branch points, it did reduce total dendrite length and thus produced similar coverage defects (Figure 1 and Figure 3).

**Figure 4:**
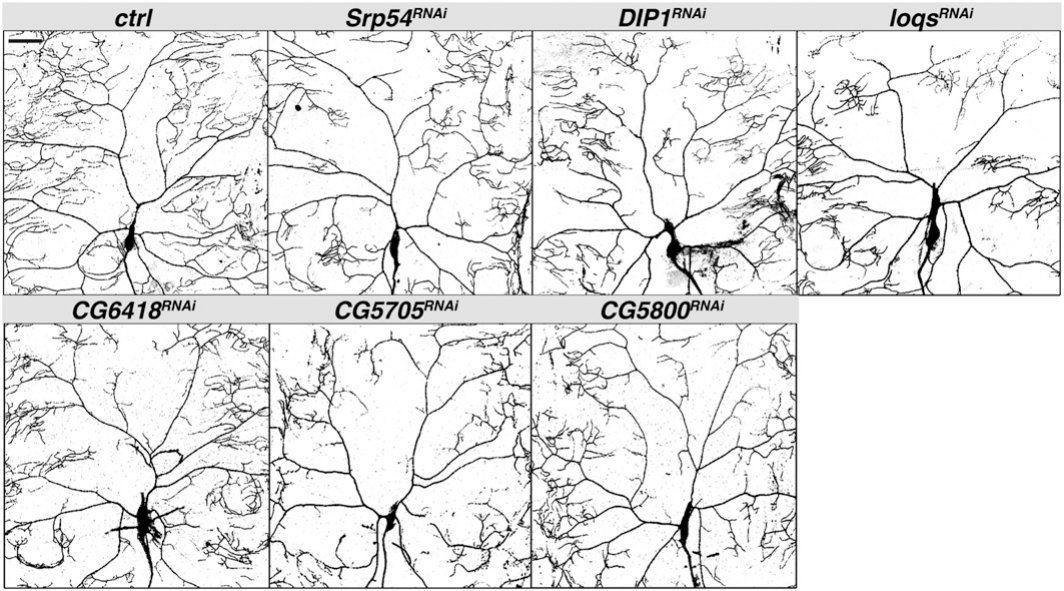
RNAi lines with branch points reduced disproportionately to dendrite length. Confocal Z-series projections of representative ddaC neurons with *ppk-GAL4* driving expression of *UAS-mcd8-GFP* alone (control) or together with the indicated *UAS-RNAi* transgene. Quantification of neurons of each genotype is shown in Figure 1. Scale bar = 50 µm. RNAi, RNA interference; UAS, upstream activating sequence.

For *Srp54*, *DISCO Interacting Protein 1* (*DIP-1*), *loquacious* (*loqs*), *CG6418*, *CG5705*, and *CG5800* RNAi, the number of branch points was reduced disproportionately to dendrite length (Figure 1 and Figure 4). Clustering of terminal branches was also observed for all but *Srp54* RNAi. Consequently, dendritic arbors appeared sparse relative to the wild-type control.

#### Altered patterning only:

Knockdown of *orb* had no discernible effect on either the number of branch points or overall dendrite length (Figure 1). However, the pattern of the dendritic tree appeared markedly different from that of wild-type control neurons, with terminal branches clustered, often toward the ends of main branches rather than being distributed throughout the tree. This aberrant pattern, with gaps in coverage, resembled the pattern observed for *x16*, *U2af38*, *snf*, and *elav* RNAi (Figure 2).

### Sensitivity of dendrite morphogenesis to translation initiation factors

**Figure 5:**
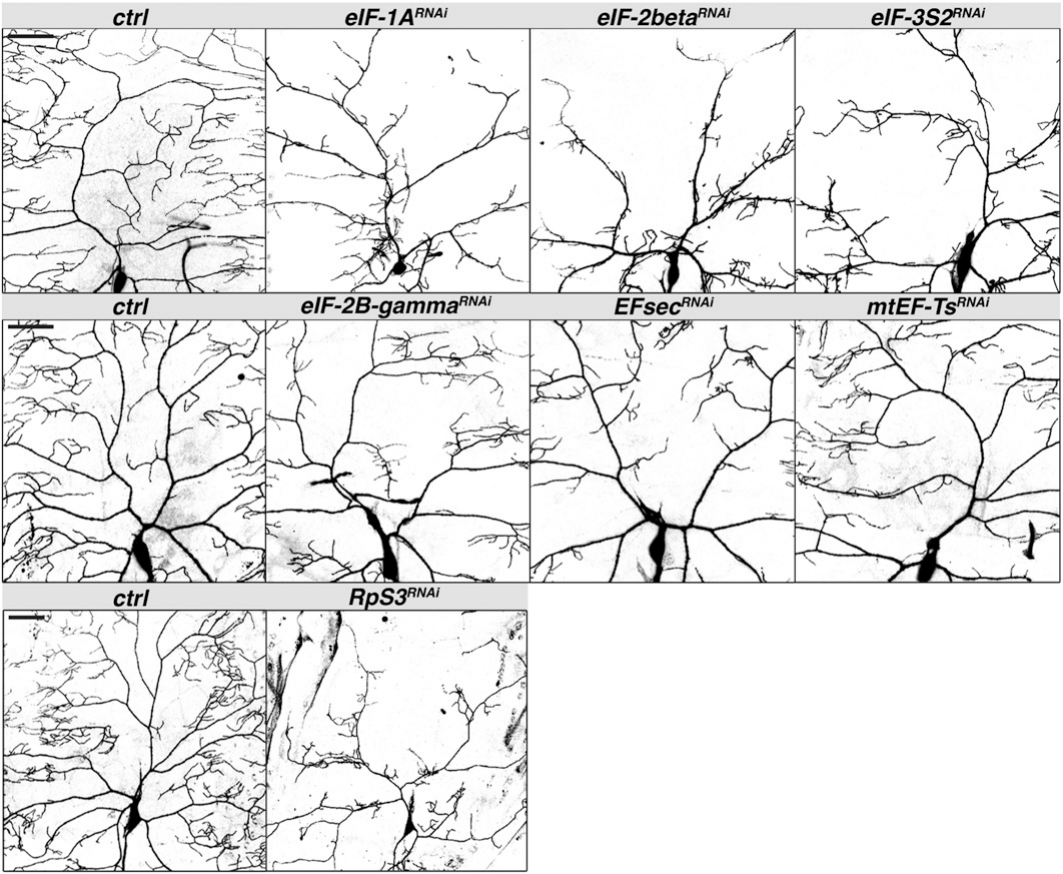
Knockdown of translation factors produces severe dendritic defects. Confocal Z-series projections of representative ddaC neurons with *GAL4^477^* (top two rows) or *ppk-GAL4* (bottom row) driving expression of *UAS-mcd8-GFP* alone (control) or together with the indicated *UAS-RNAi* transgene. Quantification for *RpS3^RNAi^* is shown in Figure 1. Scale bars = 50 µm. RNAi, RNA interference; UAS, upstream activating sequence.

Among the various types of proteins identified by this screen for roles in dendrite morphogenesis, translation initiation factors and other components of the translation machinery were highly enriched. RNAi-mediated knockdown of genes encoding initiation factors proved to be highly deleterious. The most severe effects were observed for *eIF-1A* and genes for several eIF2 and eIF3 subunits (*eIF-2alpha*, *eIF-2beta*, *eIF3-S2*, *eIF3-S4*, *eIF3-S5*, *eIF3-S8*, and *eIF3-S9*), which produced characteristic trees with truncated main branches and few, short higher order branches. Less severe but consistent phenotypes were observed for an additional eIF3 subunit gene, *eIF3-p40*, as well as for subunits of eIF2B (*eIF2B-delta*, *eIF2B-epsilon*, *eIF2B-gamma*), *eIF4a*, and *eIF4E-4* (Figure 5 and data not shown).

Although knockdown of initiation factor genes tended to result in severely decreased branching and field coverage, knockdown of translation elongation and termination factor genes (*eEf1delta*, *Ef1alpha48D*, *Ef1alpha100E*, *Ef1beta*, *Efsec*, *mtEF-Ts*, *eRF1)* generally produced milder phenotypes (Figure 5 and data not shown). This difference could reflect redundancy among elongation factors. Alternatively, because translational initiation is rate-limiting and offers the greatest opportunity for regulation, it may be more sensitive to perturbation, causing more severe phenotypic defects than disrupting translation elongation.

Knockdown of *RpS3*, which encodes a 40S ribosomal subunit protein that also has DNA repair activity and contains a K homology (KH) RNA binding domain, resulted in a phenotype resembling that of translation initiation factors. A severe branching deficit and even greater effect on dendrite length was produced by *RpS3* RNAi, leaving truncated and denuded branches (Figure 1 and Figure 5). Consistent with the similar effects produced by knockdown of translation initiation factors and *RpS3* RNAi, RpS3 resides within the domain of the 40S ribosome where translation is initiated. Taken together these results highlight the importance of translation machinery in dendrite branch formation and patterning.

## Discussion

The size, complexity, and branching morphology of the dendritic tree impact the ability of a neuron to receive inputs and are tailored according to the functions of different neurons. The elaboration of dendritic trees is a complex process involving regulated dendrite outgrowth and retraction events that determine the size, shape, and pattern of the arbor, which then must be maintained (Kulkarni and Firestein 2012). These events often occur far from the cell body and are thus likely to involve local control of protein expression in the dendrites as well as regulation of mRNA metabolism in the soma. Through tissue-specific RNAi knockdown, we have uncovered roles for an unprecedented number of highly conserved RBPs and translation factors in dendrite development.

Our RNAi screen inevitably underestimates the extent of RBP involvement due to (1) the existence of a potentially large number of RBPs without annotated RBDs (Castello *et al.* 2012); (2) RBPs not represented in existing *UAS-RNAi* collections; and (3) the incomplete knockdown associated with RNAi. Indeed, while mutational analysis has shown roles for *pum* and *dfmr1* in class IV da neuron dendrite morphogenesis (Lee *et al.* 2003; Ye *et al.* 2004; Bianco *et al.* 2010; Olesnicky *et al.* 2012), neither *pum* nor *dfmr1* RNAi had an effect. Similarly RNAi knockdown of *staufen* (*stau*), which encodes a double-stranded RNA binding protein, did not result in any overt dendritic defects whereas dendritic branching was reduced in *stau* mutant larvae (data not shown). Nonetheless, the screen was ultimately successful in identifying many RBPs and translation factors not previously known to function in dendrite morphogenesis. Moreover, we found that certain classes of RBPs or translation factors were associated with particular phenotypes, suggesting that specific points of mRNA regulation may influence distinct dendrite morphogenetic processes.

### RNA splicing and control of dendrite branch number

The generation of protein diversity through alternative splicing is prevalent in the nervous system (Li *et al.* 2007). Knockdown of three genes encoding splicing factors, *mbl*, *x16*, and *U2af38*, uniquely resulted in excessive branching. Mbl and X16, respectively, are members of the conserved muscleblind-like and serine/arginine-rich (SR) splicing factor families involved in alternative splicing (Vorbruggen *et al.* 2000; Wang *et al.* 2012). U2af38, a core spliceosomal component involved in 3′ splice site selection, has been shown to affect alternative splicing of the *Drosophila Down syndrome cell adhesion molecule* (*Dscam*) mRNA in a cell culture assay (Park *et al.* 2004) and is predicted to interact with X16 based on analysis of the vertebrate orthologs (Murali *et al.* 2011). For two additional genes encoding splicing factors, *elav* and *snf*, branch number was not affected, but overall dendrite length was decreased, leading to an elevated ratio of branch points to dendrite length similarly to *mbl*, *x16*, and *U2af38* (Figure 1). Like U2af38, Snf is a core spliceosome component that is also involved in alternative splicing of specific transcripts (Park *et al.* 2004). Elav is a neuron-specific RBP that mediates alternative splicing of several transcripts, including *neuroglian*, producing a neuron-specific Neuroglian isoform (Lisbin *et al.* 2001). Thus, a limiting step in the regulation of branch number and its coordination with the size of the neuron may be alternative splicing of one or more target mRNAs. Intriguingly, Mbl has been implicated in subcellular localization as well as splicing of its target mRNAs (Wang *et al.* 2012), suggesting that in da neurons it could target particular mRNA splicing isoforms to dendrites.

### Severity of translation initiation factor RNAi

Among the most severe phenotypes observed in the screen were those associated with translation initiation factors. Knockdown of eight subunits from three initiation factors, eIF1, eIF2, and eIF3, produced da neurons characterized by dramatic loss and shortening of branches of all orders and consequent field coverage defects. These defects may result from the conglomerate effect of a generalized decreased translation on multiple cellular functions required to generate and/or maintain the dendritic tree. In this regard, Vanishing white matter, an inherited leukoencephalopathy caused by mutations in eIF2B, is proposed to be caused by the activation of the stress response in glia due to decreased protein synthesis (Bugiani *et al.* 2010).

Alternatively, the severity of the defects could reflect a high demand for local translation in dendrite growth and remodeling, possibly in response to epidermal or neuronal cues. Seven of the translation factors we identified as positive have been shown or are predicted to be associated with microtubules (Table 2 and Table S3), suggesting that they may be transported to dendrites. Consistent with this idea, initiation factors including eIF2-alpha and eIF2-beta, both of which were positive in our screen, have been identified as components of neuronal transport ribonucleoprotein granules (Kanai *et al.* 2004). Such local translation is critical for axon growth cone navigation and for activity-dependent regulation of synaptic structure and function. Activity-dependent translation in dendrites that is required for synaptic plasticity is regulated largely at the level of initiation, through the extracellular signal-regulated kinase and mammalian target of rapamycin signaling pathways that affect the activity of initiation factors like eIF2 and eIF4E as well as elongation factors (Klann and Dever 2004; Swiech *et al.* 2008). Regulation of protein synthesis through phosphorylation of ribosomal S6 kinase (S6K) and eIF4E-binding protein by the PI3K–Akt–mTOR pathway has also been implicated in dendrite growth, branching, and spine morphogenesis in cultured hippocampal neurons (Jaworski *et al.* 2005; Kumar *et al.* 2005). Strikingly, mutation of *Tor* or *S6K* produced a dramatic decrease in class IV da neuron branch number and length and a concomitant field coverage defect (Koike-Kumagai *et al.* 2009), similar to what we observed for knockdown of initiation and elongation factors. Identifying the transcripts whose translation is altered by these perturbations will provide insight into cellular mechanisms underlying dendrite morphogenesis.

**Table 2:** Molecular functions associated with positive genes

Molecular Function	Number of Genes (%)
Translation initiation	16 (18)
Translation elongation	6 (7)
Translational repression	5 (6)
Translation termination and release	3 (3)
mRNA splicing	17 (19)
Cell death and engulfment	8 (9)
Cytoskeleton	16 (18)

RBP genes and translational factors that are required for da dendrite morphogenesis are associated with several molecular functions. The number of genes (and percentage out of the 88 positive genes and 4 positive controls) associated with each molecular function based on gene ontology terms (see Table S2) are indicated. The following Gene Ontology (GO) term accession numbers were used to group the genes by molecular function: translation initiation: GO: 0006413; translation elongation: GO: 0006414; translational repression: GO: 0030371, GO: 0000900, GO: 0017148; translation termination and release: GO: 0006415, GO: 0003747; mRNA splicing: GO: 000398, GO: 000381, GO: 0000245; cell death: GO: 0006911, GO: 0048102, GO: 0043524, GO: 0043066; cytoskeleton: GO: 0000022, GO: 0007052, GO: 0005875, GO: 0030837, GO: 0008103, GO: 0016325, GO: 0005200, GO: 0007016. mRNA; messenger RNA; RBP, RNA-binding protein.

### Link between translation factors and RBPs required for dendrite morphogenesis and cell death pathways

Eight of the positive genes have been previously implicated in cell death or engulfment (Table 2 and Table S3), suggesting that the RBPs they encode may regulate effectors of these pathways in da neurons. During pupariation, *Drosophila* class IV da neuron dendrites are eliminated through a pruning process in preparation for the formation of new projections necessary for the adult nervous system. This dendritic pruning involves dendrite severing in response to hormonal stimulation, local caspase activation in dendrites, and engulfment by phagocytes (Williams and Truman 2005; Kuo *et al.* 2006; Williams *et al.* 2006). Confinement of caspase activity to dendrites promotes their elimination while protecting the neuron from apoptotic cell death (Kuo *et al.* 2006; Williams *et al.* 2006). We have previously shown that the Nos/Pum translational repression complex is required to maintain the dendritic complexity of class IV da neurons in part by repressing expression of the proapoptotic protein Hid, which promotes caspase activation (Olesnicky *et al.* 2012). The identification of multiple RBPs with links to cell death/engulfment whose depletion resulted in decreased branching suggests that they may be part of a concerted effort to prevent inappropriate activation of the pruning pathway prior to pupariation. Alternatively, nonapoptotic functions of the cell death/engulfment machinery may be involved in promoting the branch retraction that normally occurs during sculpting of the dendritic arbor. These RBPs may serve to modulate expression of components of the cell death/engulfment machinery, achieving a balance between dendrite growth and retraction necessary for proper branching morphogenesis.

Interestingly, many apoptotic genes are alternatively spliced, often to yield protein isoforms with antagonistic pro- and anti-apoptotic functions (Schwerk and Schulze-Osthoff 2005). Thus, the regulation of alternative splicing may impact dendrite morphogenesis in part through effects on apoptotic pathway activity. One of the splicing regulators identified here, Mbl, has been implicated in apoptosis in wing imaginal disc cells (Ho *et al.* 2004; Vicente-Crespo *et al.* 2008). Loss or switch in function of an apoptotic pathway component due to impaired alternative splicing could shift the balance between growth and retraction to produce the excess branching phenotype we observed in *mbl* RNAi neurons.

### Redeployment of RBPs from germline to nervous system

The majority of *Drosophila* RBPs are maternally deposited in the embryo (Tomancak *et al.* 2002) and consistent with this, 86% of the RBPs identified to function in da neurons are expressed above “low” levels in the *Drosophila* ovary according to FlyAtlas Anatomical Expression Data (Chintapalli *et al.* 2007) or modENCODE Tissue Expression Data (http://www.modencode.org/celniker/). In addition, 76% of RBPs with da neuron functions are expressed in the larval central nervous system, including 82% of those with ovarian expression. Both oocytes and neurons may share an elevated demand for post-transcriptional gene regulation compared to other cell types. Early developmental events in *Drosophila* rely on maternally expressed genes, which is thought to allow for rapid embryonic development (Kalinka and Tomancak 2012). Thus, post-transcriptional mechanisms are the only means for temporal and spatial regulation of gene expression during this time. In particular, mRNA localization and local translation are heavily used in the establishment of regional differences within the oocyte and early embryo that are necessary for formation of the body axes and the specification of the germline. Similarly, the need for rapid and local responses of da neuron dendrites to cues from their epidermal substratum or other dendrites during larval growth may best be served by post-transcriptional mechanisms. At least eight of the genes identified in the screen (*aret*, *brat*, *4EHP*, *orb*, *osk*, *rump*, *spoon*, *sqd*) as well as *dfmr1*, *glo*, *nos*, *pum*, *stau*, and *smg* have been shown to play roles in establishing asymmetries in the oocyte and early embryo by regulating the localization or local translation of target mRNAs (References in FlyBase gene reports: http://flybase.org). Moreover, both *nos* and *osk* mRNAs are transported into da neuron dendrites, consistent with a local regulatory function for Nos and Osk proteins (Brechbiel and Gavis 2008; Xu *et al.* 2013). The redeployment of such factors in neurons highlights the contribution of post-transcriptional control to neurogenesis.

## Supplementary Material

Supporting Information
