# Supplementary material for: Extensive Use of RNA-Binding Proteins in Drosophila Sensory Neuron Dendrite Morphogenesis
Source: G3 (Bethesda). 2013 Dec 17;4(2):297–306. doi: 10.1534/g3.113.009795 (PMC3931563; doi:10.1534/g3.113.009795)
Supplement: Supporting Information [file supp_4_2_297__index.html]

Extensive Use of RNA Binding Proteins in Drosophila Sensory Neuron Dendrite Morphogenesis — Extensive Use of RNA-Binding Proteins in Drosophila Sensory Neuron Dendrite Morphogenesis — Supporting Information 

# Extensive Use of RNA-Binding Proteins in *Drosophila* Sensory Neuron Dendrite Morphogenesis

## Supporting Information for Olesnicky *et al.*, 2014

**Files in this Data Supplement:**

- Supporting Information - Tables S1-S3 (PDF, 837 KB)
- Table S1 - List of RNAi stocks screened. (PDF, 312 KB)
- Table S2 - Homologs of *Drosophila* RBPs and translational factors with functions in dendrite morphogenesis. (PDF, 632 KB)
- Table S3 - The gene ontology (GO) terms for the RBPs and Translational Factors that are required for dendrite morphogenesis in *Drosophila* da neurons were found by searching The Gene Ontology website (www.geneontology.org database release May 26, 2012). (PDF, 594 KB)
