# Supplementary material for: Extensive Use of RNA-Binding Proteins in Drosophila Sensory Neuron Dendrite Morphogenesis
Source: G3 (Bethesda). 2013 Dec 17;4(2):297–306. doi: 10.1534/g3.113.009795 (PMC3931563; doi:10.1534/g3.113.009795)
Supplement: Supporting Information [file supp_g3.113.009795_009795SI.pdf]

G3: Genes | Genomes | Genetics

Extensive use of RNA binding proteins in *Drosophila* sensory neuron dendrite morphogenesis

Eugenia C. Olesnicky[1,2], Darrell J. Killian[3], Evelyn Garcia[2], Mary C. Morton[2], Alan R. Rathjen[2], Ismail E. Sola[2] and Elizabeth R. Gavis[1*]

[1] Department of Molecular Biology, Princeton University, Princeton, NJ 08544
[2] Department of Biology, University of Colorado at Colorado Springs, Colorado Springs, CO 80918
[3] Biology Department, The Colorado College, Colorado Springs, CO 80903

[*]Corresponding author
Elizabeth R. Gavis
Department of Molecular Biology
Washington Road
Princeton University
Princeton, NJ 08544
(609) 258-3857

gavis@princeton.edu

DOI: 10.1534/g3.113.009795

Table S1 List of RNAi stocks screened.

VDRC STOCK NUMBERS

10192	100563	103141	104978	106257
13305	100611	103355	105054	106318
17003	100693	103365	105107	106321
17065	100702	103411	105121	106393
20321	100709	103427	105135	106475
21379	100722	103665	105148	106636
21763	100723	103704	105254	106645
22837	100732	103708	105271	106696
22846	100739	103728	105291	106734
23677	100775	103735	105322	106754
24725	100805	103751	105325	106762
24889	100813	103769	105437	106833
26045	100817	103789	105486	106944
26243	101060	103791	105495	106972
26472	101288	103913	105500	106994
27752	101399	103940	105543	107004
27776	101412	103972	105585	107007
28072	101435	104096	105612	107013
29116	101465	104156	105619	107031
31324	101508	104160	105672	107063
31364	101513	104183	105704	107064
34210	101537	104187	105763	107112
34711	101555	104253	105825	107147
35288	101567	104327	105883	107153
37863	101740	104334	105907	107268
40352	101765	104342	105907	107282
40683	101765	104351	105949	107304
44895	101781	104379	105950	107385
45027	101925	104401	105954	107445
47973	102118	104403	105963	107459
49506	102159	104471	105991	107575
50094	102173	104481	105992	107593
100001	102360	104502	106041	107595
100226	102360	104562	106047	107709
100291	102442	104715	106078	107723
100310	102597	104865	106079	107750
100356	102736	104876	106189	107829
100546	102825	104941	106240	107953

107958	108216	108900	109689	110143
107993	108310	108916	109739	110165
107993	108351	108933	109742	110357
107999	108358	108947	109762	110410
108026	108376	108950	109762	110441
108065	108552	108993	109782	110451
108072	108580	109212	109796	110452
108072	108642	109221	109892	110457
108083	108653	109436	109911	110472
108094	108666	109465	109951	110476
108158	108734	109500	110008	110514
108169	108828	109602	110075	110518
108186	108847	109672	110102	

NIG STOCK NUMBERS

1316R-1	4886R-1	7185R-2	11454R-4	17697R-1
2931R-3	4887R-3	7697R-1	11505R-2	17838R
3056R-1	5263R-2	7879R-1	11726R-1	18179
3335R-1	5439R-1	7903R-1	14035R-1	18259R-3
4035R-1	5808R-1	8933	14230R-1	20839
4119R-2	6946R-1	9346R-1	14641R-1	27752
4262R-4	6961R-3	10868R-1	14891R-2	32169R-2
4824R-5	6995R-2	10948R-3	14900R-1	45815
4878R-3	6999R-3	11266R-3	17540-R	49473

TRiP (Bloomington) STOCK NUMBERS

25970	28036	30518
26293	28049	34646
26745	28071	
27040	28314	
27051	28360	
27320	28371	
27484	28566	
27565	28590	
27659	28931	
27704	28991	

OTHER

UAS-nosRNAi (Menon et al. 2002)

UAS-pumRNAi (Menon et al. 2002)

Table S2 Homologs of *Drosophila* RBPs and translational factors with functions in dendrite morphogenesis

Drosophila CG	*Drosophila* Protein Name	BLAST - Fly to Human	Human Homolog	BLAST - Fly to Mouse*	Mouse Homolog	BLAST - Fly to *C. elegans*	*C. elegans* Homolog
CG1873	Elongation factor 1 alpha 100E (EF1 alpha 100E)	0	elongation factor 1-alpha 1 (NP_001393)	0	elongation factor 1-alpha 1 (NP_034236)	0	EEF-1A.2 (R03G5.1a)/EEF-1A.1 (F31E3.5)
CG3056	Sister-of-Sex-lethal (Ssx)	6e-53	ELAV-like protein 4 (NP_001138246)	3e-53	ELAV-like protein 4 (NP_001156869)	1e-47	EXC-7 (F35H8.5)
CG3249	Spoonbill (Spoon)/yu	1e-46	A-kinase anchor protein 1 (NP_003479)	3e-46	A-kinase anchor protein 1 (NP_001036006)	3e-30	C56G2.1
CG3582	U2 small nuclear riboprotein auxiliary factor 38 (U2AF38)	3e-119	splicing factor U2AF 35 kDa subunit (NP_001020374)	3e-119	splicing factor U2AF 35 kDa subunit (NP_001157241)	9e-90	UAF-2 (Y116A8C.35)
CG3806	eIF2B-ε	2e-69	translation initiation factor eIF-2B subunit epsilon (NP_003898)	5e-98	translation initiation factor eIF-2B subunit epsilon (NP_758469)	3e-22	D2085.3
CG4119		2e-47	RNA-binding protein 25 (NP_067062)	2e-47	RNA-binding protein 25 (NP_081625)	8e-28	W04D2.6
CG4152	Lethal(2)35Df (L(2)35Df)	0	superkiller viralicidic activity 2-like 2 (NP_056175)	0	superkiller viralicidic activity 2-like 2 (NP_082427)	0	MTR-4 (W08D2.7)
CG4153	Eukaryotic initiation factor 2β (eIF-2β)	4e-101	eukaryotic translation initiation factor 2 subunit 2 (NP_003899)	2e-102	eukaryotic translation initiation factor 2 subunit 2 (NP_080306)	1e-72	ITFB-1 (K04G2.1)
CG4262	Embryonic lethal abnormal vision (Elav)	9e-126	ELAV-like protein 4 (NP_001138246	7e-126	ELAV-like protein 4 (NP_001156869)	3e-75	EXC-7 (F35H8.5)
CG4396	Found in neurons (Fne)	2e-146	ELAV-like protein 2 (NP_001164666)	7e-147	ELAV-like protein 4 (NP_001156869)	4e-85	EXC-7 (F35H8.5)
CG4528	Sans fille (Snf)	3e-103	U2 small nuclear ribonucleoprotein B (NP_003083)	1e-101	U2 small nuclear ribonucleoprotein B (NP_067310)	5e-70	RNP-3 (K08D10.3)
CG4602	Srp54	2e-55	splicing regulatory glutamine/lysine-rich protein 1 (NP_001070667)	7e-30	splicing factor, arginine/serine-rich 11 (NP_001087221)	8e-45	RSP-7 (D2089.1)

CG4792	Dicer-1 (Dcr-1)	3e-122	endoribonuclease Dicer (NP_001182502)	2e-123	endoribonuclease Dicer (NP_683750)	3e-124	DCR-1 (K12H4.8)
CG4878	eIF3-S9	0	eukaryotic translation initiation factor 3 subunit B (NP_003742)	0	eukaryotic translation initiation factor 3 subunit B (NP_598677)	6e-114	EIF-3.b (Y54E2A.11)
CG4886	Cyclophilin-33 (Cyp33)	9e-146	peptidyl-prolyl cis-trans isomerase E (NP_006103)	2e-144	peptidyl-prolyl cis-trans isomerase E (NP_062362)	5e-124	CYN-13 (Y116A8C.34)
CG4887		4e-98	RNA-binding protein 10 (NP_001191397)	2e-99	RNA-binding protein 10 (NP_663602)	2e-23	T08B2.5
CG4912	eEF1δ	2e-67	elongation factor 1-delta (NP_001951)	4e-66	elongation factor 1-delta (NP_075729)	6e-40	EEF-1b.1 (F54H12.6)
CG4954	eIF3-S8	0	eukaryotic translation initiation factor 3 subunit C (NP_003743)	0	Eukaryotic translation initiation factor 3, subunit C (NP_666312)	0	EIF-3.c (T23D8.4)
CG5168		6e-163	WD repeat and FYVE domain-containing protein 2 (NP_443182)	8e-163	WD repeat and FYVE domain-containing protein 2 (NP_780755)	2e-98	WDFY-2 (D2013.2)
CG5263	Smaug (Smg)	9e-24	Smaug homolog 2 (NP_060498)	3e-24	smaug homolog 1 (NP_001156905)	7e-17	ZC190.4
CG5439		5e-30	sorting nexin-29 (NP_115543)	4e-29	sorting nexin-29 (NP_083240)	2e-08	F13E9.1
CG5589		8e-171	probable ATP-dependent RNA helicase DDX52 (NP_008941)	5e-60	probable ATP-dependent RNA helicase DDX23 (NP_001074450)	6e-112	R05D11.4
CG5605	Eukaryotic release factor 1 (eRF1)	0	eukaryotic peptide chain release factor subunit 1 (NP_004721)	0	eukaryotic peptide chain release factor subunit 1 (NP_659115)	0	T05H4.6
CG5637	Nanos (Nos)	1e-18	nanos homolog 1 (NP_9556319)	5e-19	nanos homolog 1 (NP_848508)	1e-07	NOS-1 (R03D7.7)
CG5705		4e-90	peptide chain release factor 1-like, mitochondrial isoform a (NP_061914)	6e-94	peptide chain release factor 1-like, mitochondrial precursor (NP_780583)	1e-67	W03F8.3
CG5800		0	probable ATP-dependent RNA	0	probable ATP-dependent RNA	1e-172	Y23H5B.6

			helicase DDX10 (NP_004389)		helicase DDX10 (NP_084212)		
CG6341	Elongation factor 1 β (Ef1β)	3e-78	elongation factor 1-beta (NP_001950)	8e-84	elongation factor 1-beta (NP_061266)	5e-63	EEF-1b.1 (F54H12.6)
CG6375	Pitchoune (Pit)	0	ATP-dependent RNA helicase DDX18 (NP_006764)	0	ATP-dependent RNA helicase DDX18 (NP_080136)	1e-175	B0511.6
CG6412	mtEF-Ts	6e-55	elongation factor Ts, mitochondrial isoform 2 precursor (NP_005717.3)	7e-60	elongation factor Ts, mitochondrial precursor (NP_079813)	9e-52	TSFM-1 (F55C5.5)
CG6418		0	ATP-dependent RNA helicase DDX42 (NP_031398)	0	ATP-dependent RNA helicase DDX42 (NP_082350)	0	C46F11.4
CG6493	Dicer-2 (Dcr-2)	6e-101	endoribonuclease Dicer (NP_001182502)	3e-103	endoribonuclease Dicer (NP_683750)	3e-70	DCR-1 (K12H4.8)
CG6539	Gemin3 (Gem3)	3e-77	probable ATP-dependent RNA helicase DDX20 (NP_009135)	7e-78	probable ATP-dependent RNA helicase DDX20 (NP_059093)	2e-67	CGH-1 (C07H6.5)
CG6779	Ribosomal protein S3 (RpS3)	1e-139	40S ribosomal protein S3 (NP_000996)	6e-140	40S ribosomal protein S3 (NP_036182)	6e-124	RPS-3 (C23G10.3)
CG6866	Loquacious (Loqs)	2e-49	interferon-inducible double stranded RNA-dependent protein kinase activator A (NP_001132990)	9e-50	interferon-inducible double stranded RNA-dependent protein kinase activator A (NP_036001)	6e-09	D1037.1
CG6946	Glorund (Glo)	9e-50	heterogeneous nuclear ribonucleoprotein F (NP_004957)	1e-49	heterogeneous nuclear ribonucleoprotein F (NP_598595)	3e-32	SYM-2 (ZK1067.6)
CG6961		6e-13	polymerase delta-interacting protein 3 (NP_835237)	1e-12	polymerase delta interacting protein 3 (NP_848742)	No Hit	No Hit
CG6987	SF2	1e-81	serine/arginine-rich splicing factor 1 (NP_008855)	3e-76	serine/arginine-rich splicing factor 1 (NP_001071635)	1e-70	RSP-3 (Y111B2A.18)
CG7082	Papi	7e-53	tudor and KH domain-containing protein (NP_006853)	2e-51	tudor and KH domain-containing protein (NP_082583)	1e-42	C56G2.1

CG7269	Helicase at 25E (Hel25E)	0	spliceosome RNA helicase DDX39B (NP_004631)	0	spliceosome RNA helicase Ddx39b (NP_062667)	0	HEL-1 (C26D10.2)
CG7903		2e-15	RNA-binding protein 14 (NP_006319)	7e-15	RNA-binding protein 14 (NP_063922)	No Hit	No Hit
CG8053	Eukaryotic inititation factor 1A (eIF-1A)	3e-75	eukaryotic translation initiation factor 1A, X-chromosomal (NP_001403)	1e-74	eukaryotic translation initiation factor 1A (NP_034250)	2e-63	EIF-1.a (H06H21.3)
CG8190	eIF2Bgamma	3e-86	translation initiation factor eIF-2B subunit gamma (NP_065098)	2e-91	eukaryotic translation initiation factor 2B, subunit 3 gamma (NP_001104747)	1e-39	PPP-1 (C15F1.4)
CG8205	Fusilli (Fus)	6e-150	epithelial splicing regulatory protein 1 (NP_001116297)	7e-150	epithelial splicing regulatory protein 1 (NP_918944)	5e-82	SYM-2 (ZK1067.6)
CG8280	Elongation factor 1 alpha 48D (EF1 alpha 48D)	0	elongation factor 1-alpha 1 (NP_001393)	0	elongation factor 1-alpha 1 (NP_034236)	0	EEF-1A.2 (R03G5.1a)/EEF-1A.1 (F31E3.5)
CG8636	eIF3-S4	1e-70	eukaryotic translation initiation factor 3 subunit G (NP_003746)	1e-70	eukaryotic translation initiation factor 3 subunit G (NP_058572)	4e-44	EIF-3.g (F22B5.2)
CG8730	Drosha	0	ribonuclease 3 (NP_001093882)	0	ribonuclease 3 (NP_081075)	0	DRSH-1 (F26E4.10)
CG8882	Trip1/eIF3-S2	2e-149	eukaryotic translation initiation factor 3 subunit I (NP_003748)	5e-149	eukaryotic translation initiation factor 3 subunit I (NP_061269)	4e-109	EIF-3.i (Y74C10AR.1)
CG9054	Dead-box-1 (Ddx1)	0	ATP-dependent RNA helicase DDX1 (NP_004930)	0	ATP-dependent RNA helicase DDX1 (NP_598801)	0	Y55F3BR.1
CG9075	Eukaryotic initiation factor 4a (eIF-4a)	0	eukaryotic initiation factor 4A-II (NP_001958)	0	eukaryotic initiation factor 4A-III (NP_619610)	0	INF-1 (F57B9.6)
CG9107		4e-48	ribosomal RNA-processing protein 7 homolog A (NP_056518)	6e-51	ribosomal RNA-processing protein 7 homolog A (NP_083377)	1e-11	ZC434.3
CG9124	Eukaryotic initiation factor 3 p40 (eIF-3p40)	3e-99	eukaryotic translation initiation factor 3 subunit H (NP_003747)	1e-100	eukaryotic translation initiation factor 3 subunit H (NP_542366)	1e-49	EIF-3.h (C41D11.2)

CG9218	Smooth (Sm)	2e-100	Heterogeneous nuclear ribonucleoprotein L (NP_001005335)	3e-99	Heterogeneous nuclear ribonucleoprotein L (NP_796275)	3e-69	C44B7.2
CG9373	Rumpelstiltskin (Rump)	2e-25	Myelin expression factor 2 (NP_034982.2)	9e-47	Myelin expression factor 2 (NP_057216.2)	1e-46	C25A1.4
CG9412	Rasputin (Rin)	3e-47	ras GTPase-activating protein-binding protein 2 (NP_987100)	4e-47	ras GTPase-activating protein-binding protein 2 (NP_035946)	3e-10	K08F4.2
CG9680	Dead box protein 73D (Dbp73D)	2e-107	ATP-dependent RNA helicase DDX51 (NP_778236)	8e-106	ATP-dependent RNA helicase DDX51 (NP_081432)	4e-59	ZK686.2
CG9755	Pumilio (Pum)	0	pumilio homolog 2 (NP_056132)	0	Pumilio homolog 2 (NP_001153694)	6e-128	PUF-9 (W06B11.2)
CG9769	eIF3-S5	3e-94	eukaryotic translation initiation factor 3 subunit F (NP_003745)	4e-93	eukaryotic translation initiation factor 3 subunit F (NP_079620)	4e-47	EIF-3.f (D2013.7)
CG9809	Spargel	3e-24	peroxisome proliferator-activated receptor gamma coactivator-related protein 1 (NP_055877)	2e-24	peroxisome proliferator-activated receptor gamma coactivator-related protein 1 (NP_001074683)	2e-05	RPN-7 (K04G7.10)
CG9841	EfSec	1e-146	selenocysteine-specific elongation factor (NP_068756)	7e-151	selenocysteine-specific elongation factor (NP_075547)	2e-68	SELB-1 (C47B2.7)
CG9946	Eukaryotic translation initiation factor 2α (eIF- 2α)	4e-134	eukaryotic translation initiation factor 2 subunit 1 (NP_004085)	2e-134	eukaryotic translation initiation factor 2 subunit 1 (NP_080390)	2e-108	Y37E3.10
CG10084	Second mitotic wave missing (Swm)	4e-51	RNA-binding protein 26 (NP_071401)	5e-52	RNA-binding protein 26 (NP_598838)	3e-16	B0336.3
CG10124	eIF4E-4	6e-63	Eukaryotic translation initiation factor 4E (NP_001959)	2e-63	eukaryotic translation initiation factor 4E (NP_031943)	2e-54	IFE-3 (B0348.6)
CG10203	X16	7e-35	serine/arginine-rich splicing factor 3 (NP_003008)	2e-34	serine/arginine-rich splicing factor 7 (NP_001026854)	2e-25	RSP-6 (C33H5.12b)

CG10315	eIF2B-δ	3e-123	translation initiation factor eIF-2B subunit delta (NP_001029288)	1e-123	translation initiation factor eIF-2B subunit delta (NP_034252)	5e-22	F11A3.2
CG10466		7e-66	RNA-binding motif protein, X-linked 2 (NP_057108)	3e-65	RNA-binding motif protein, X-linked 2 (NP_775552)	2e-48	C30B5.4
CG10719	Brain tumor (Brat)	3e-42	brain expressed ring finger; tripartite motif-containing protein 3 (NP_001234936)	5e-42	tripartite motif-containing protein 3 (NP_061368)	2e-172	NCL-1 (ZK112.2)
CG10777		0	probable ATP-dependent RNA helicase DDX5 (NP_004387)	0	probable ATP-dependent RNA helicase DDX5 (NP_031866)	3e-167	F58E10.3
CG10868	Oo18 RNA binding protein (Orb)	2e-126	cytoplasmic polyadenylation element-binding protein 1 (NP_001073002)	2e-124	cytoplasmic polyadenylation element-binding protein 1 (NP_001239455)	8e-69	CPB-3 (B0414.5)
CG10881	eIF3-SG2	1e-73	eukaryotic translation initiation factor 3 subunit G (NP_003746)	6e-74	eukaryotic translation initiation factor 3 subunit G (NP_058572)	3e-43	EIF-3.g (F22B5.2)
CG10901	Oskar (Osk)	No Hit	No Hit	No Hit	No Hit	No Hit	No Hit
CG11266		8e-126	RNA-binding protein 39 (NP_001229529)	7e-124	RNA-binding protein 39 (NP_573505)	6e-84	Y55F3AM.3
CG11334		2e-142	methylthioribose-1-phosphate isomerase (NP_001026897)	4e-129	methylthioribose-1-phosphate isomerase (NP_080699)	5e-69	C01G10.9
CG11454		6e-23	RNA-binding protein 7 (NP_057174)	4e-22	RNA-binding protein 7 (NP_659197)	5e-10	SAP-49 (C08B11.5)
CG11505		3e-71	La-related protein 4 (NP_001164275)	2e-70	La-related protein 4 (NP_001074417)	1e-33	LARP-5 (T12F5.5)
CG11726		7e-10	eukaryotic translation initiation factor 4H (NP_071496)	7e-10	eukaryotic translation initiation factor 4H (NP_291039)	8e-06	HRPF-1 (W02D3.11)
CG12493		8e-06	interleukin enhancer-binding factor 3 (NP_703194)	3e-06	interleukin enhancer-binding factor 3 (NP_001036172)	No Hit	No Hit
CG13425	Bancal (Bl)	3e-40	Heterogeneous nuclear	4e-40	Heterogeneous nuclear	2e-39	F26B1.2

			ribonucleoprotein K (NP_112553)		ribonucleoprotein K (NP_079555)		
CG14718		2e-21	TATA-binding protein-associated factor 2N (NP_003478)	4e-21	TATA-binding protein-associated factor 2N (NP_081703)	2e-08	FUST-1 (C27H5.3)
CG14891		7e-07	F-box/LRR-repeat protein 13 (NP_659469)	2e-06	F-box/LRR-repeat protein 14 (NP_598701)	No Hit	No Hit
CG16901	Squid (Sqd)	5e-51	Heterogeneous nuclear ribonucleoprotein D0 (NP_002129)	6e-51	Heterogeneous nuclear ribonucleoprotein D0 (NP_031542)	3e-41	SQD-1 (Y73B6BL.6)
CG17492	Mindbomb 2 (Mib2)	0	E3 ubiquitin-protein ligase MIB2 (NP_001164158)	0	E3 ubiquitin-protein ligase MIB2 (NP_001243036)	7e-23	T28D6.4
CG17686	Disco interacting protein 1 (DIP1)	2e-15	double-stranded RNA-specific editase 1 (NP_001103)	2e-15	double-stranded RNA-specific editase 1 (NP_001020008)	No Hit	No Hit
CG18259		4e-13	polymerase delta-interacting protein 3 (NP_835237)	8e-13	polymerase delta interacting protein 3 (NP_848742)	No Hit	No Hit
CG31061	Gustatory receptor 98d (Gr98d)	No Hit	No Hit	No Hit	No Hit	No Hit	No Hit
CG31762	Arrest (Aret)/Bruno (Bru)	4e-131	CUGBP Elav-like family member 2 (NP_001020248)	3e-133	CUGBP Elav-like family member 2 (NP_001103702)	9e-118	ETR-1 (T01D1.2f)
CG32423	Alan shepard (Shep)	2e-81	RNA-binding motif, single-stranded-interacting protein 2 (NP_002889)	2e-82	RNA-binding motif, single-stranded-interacting protein 2 (NP_001034169)	5e-60	SUP-26 (R10E4.2)
CG32706		5e-16	activator of basal transcription 1 (NP_037507)	1e-16	activator of basal transcription 1 (NP_038952)	3e-11	F57B10.8
CG33100	4EHP	8e-44	eukaryotic translation initiation factor 4E type 2 (NP_004837)	1e-44	eukaryotic translation initiation factor 4E type 2 (NP_075803)	2e-36	IFE-4 (C05D9.5)
CG33106	Multiple ankyrin repeats single KH domain (Mask)	0	ankyrin repeat and KH domain-containing protein 1 (NP_060217)	0	ankyrin repeat and KH domain-containing protein 1 (NP_780584)	2e-154	R11A8.7

CG33197	Muscleblind (Mbl)	2e-42	muscleblind-like protein 1 (NP_066368)	1e-42	muscleblind-like protein 1 (NP_064391)	3e-57	MBL-1 (K02H8.1)
CG34354		1e-79	nucleolysin TIA-1 isoform p40 (NP_071505)	6e-79	nucleolysin TIA-1 (NP_035715)	1e-77	TIAR-1 (C18A3.5)
CG40351	Set1	2e-100	histone-lysine N-methyltransferase SETD1A (NP_055527)	8e-100	SET domain containing 1A (NP_821172)	2e-71	SET-2 (C26E6.9)

BLAST-P search of RBPs and translational factors that are required for dendrite morphogenesis in *Drosophila* da neurons to identify the most homologous human, mouse and *C. elegans* proteins. For *Drosophila* proteins that exist as multiple isoforms, the longest isoform was used. E values reported as 0 are less than 1e-180. “No Hit” indicates that there are no homologs with an E value greater than 1e-05.

Table S3 The gene ontology (GO) terms for the RBPs and Translational Factors that are required for dendrite morphogenesis in *Drosophila* da neurons were found by searching The Gene Ontology website (www.geneontology.org database release May 26, 2012). The GO terms are categorized as Biological Process, Cellular Component, and Molecular Function.

CG	Gene Name	Biological Process	Cellular Component	Molecular Function
CG1873	*Ef1α100E*	GO:0022008 : neurogenesis<br>GO:0006412 : translation<br>GO:0006414 : translational elongation	GO:0005737 : cytoplasm<br>GO:0005853 : eukaryotic translation elongation factor 1 complex<br>GO:0005811 : lipid particle	GO:0005525 : GTP binding<br>GO:0003924 : GTPase activity<br>GO:0003746 : translation elongation factor activity
CG3056	*ssx*			GO:0003729 : mRNA binding<br>GO:0000166 : nucleotide binding<br>GO:0003723 : RNA binding
CG3249	*spoon (yu)*	GO:0007298 : border follicle cell migration<br>GO:0060269 : centripetally migrating follicle cell migration<br>GO:0051276 : chromosome organization<br>GO:0046843 : dorsal appendage formation<br>GO:0008069 : dorsal/ventral axis specification, ovarian follicular epithelium<br>GO:0008298 : intracellular mRNA localization<br>GO:0007616 : long-term memory<br>GO:0008070 : maternal determination of dorsal/ventral axis, ovarian follicular epithelium, germ-line encoded<br>GO:0030837 : negative regulation of actin filament polymerization<br>GO:0008104 : protein localization<br>GO:0006403 : RNA localization<br>GO:0006099 : tricarboxylic acid cycle	GO:0005794 : Golgi apparatus<br>GO:0005739 : mitochondrion	GO:0051018 : protein kinase A binding<br>GO:0003723 : RNA binding
CG3582	*U2af38*	GO:0007052 : mitotic spindle organization<br>GO:0000398 : nuclear mRNA splicing, via spliceosome<br>GO:0000381 : regulation of alternative nuclear mRNA splicing, via spliceosome<br>GO:0008380 : RNA splicing	GO:0005634 : nucleus<br>GO:0071011 : precatalytic spliceosome<br>GO:0005681 : spliceosomal complex<br>GO:0005686 : U2 snRNP	GO:0003729 : mRNA binding<br>GO:0000166 : nucleotide binding<br>GO:0008187 : poly-pyrimidine tract binding<br>GO:0008270 : zinc ion binding
CG3806	*eIF2B-ε*	GO:0016070 : RNA metabolic process	GO:0005829 : cytosol	GO:0005085 : guanyl-nucleotide exchange factor activity

		GO:0006413 : translational initiation	GO:0005851 : eukaryotic translation initiation factor 2B complex	GO:0016779 : nucleotidyltransferase activity<br>GO:0003743 : translation initiation factor activity
CG4119		GO:0000398 : nuclear mRNA splicing, via spliceosome<br>GO:0000381 : regulation of alternative nuclear mRNA splicing, via spliceosome	GO:0005634 : nucleus<br>GO:0071011 : precatalytic spliceosome	GO:0003729 : mRNA binding<br>GO:0003676 : nucleic acid binding<br>GO:0000166 : nucleotide binding
CG4152	*l(2)35Df*	GO:0022008 : neurogenesis<br>GO:0000398 : nuclear mRNA splicing, via spliceosome	GO:0071013 : catalytic step 2 spliceosome	GO:0005524 : ATP binding<br>GO:0004004 : ATP-dependent RNA helicase activity<br>GO:0003676 : nucleic acid binding
CG4153	*eIF-2ß*	GO:0016199 : axon midline choice point recognition<br>GO:0001677 : formation of translation initiation ternary complex<br>GO:0000022 : mitotic spindle elongation<br>GO:0007052 : mitotic spindle organization<br>GO:0006413 : translational initiation	GO:0005829 : cytosol<br>GO:0005850 : eukaryotic translation initiation factor 2 complex<br>GO:0005875 : microtubule associated complex	GO:0005525 : GTP binding<br>GO:0003743 : translation initiation factor activity<br>GO:0000049 : tRNA binding
CG4262	*elav*	GO:0007417 : central nervous system development<br>GO:0031441 : negative regulation of mRNA 3'-end processing<br>GO:0007399 : nervous system development<br>GO:0016070 : RNA metabolic process	GO:0015030 : Cajal body<br>GO:0005634 : nucleus	GO:0003729 : mRNA binding<br>GO:0000166 : nucleotide binding<br>GO:0008266 : poly(U) RNA binding<br>GO:0003723 : RNA binding
CG4396	*fne*	GO:0051252 : regulation of RNA metabolic process	GO:0005737 : cytoplasm	GO:0003729 : mRNA binding<br>GO:0000166 : nucleotide binding<br>GO:0008266 : poly(U) RNA binding
CG4528	*snf*	GO:0019099 : female germ-line sex determination<br>GO:0000398 : nuclear mRNA splicing, via spliceosome<br>GO:0048477 : oogenesis<br>GO:0007539 : primary sex determination, soma	GO:0071013 : catalytic step 2 spliceosome<br>GO:0005634 : nucleus<br>GO:0071011 : precatalytic spliceosome<br>GO:0043234 : protein complex	GO:0003729 : mRNA binding<br>GO:0000166 : nucleotide binding<br>GO:0005515 : protein binding<br>GO:0035614 : snRNA stem-loop binding

		GO:0000381 : regulation of alternative nuclear mRNA splicing, via spliceosome<br>GO:0008380 : RNA splicing	GO:0030532 : small nuclear ribonucleoprotein complex<br>GO:0005681 : spliceosomal complex<br>GO:0005685 : U1 snRNP<br>GO:0005692 : U11 snRNP<br>GO:0005686 : U2 snRNP	GO:0030619 : U1 snRNA binding<br>GO:0030620 : U2 snRNA binding
CG4602	*Srp54*	GO:0006376 : mRNA splice site selection<br>GO:0022008 : neurogenesis<br>GO:0000398 : nuclear mRNA splicing, via spliceosome<br>GO:0000381 : regulation of alternative nuclear mRNA splicing, via spliceosome	GO:0071013 : catalytic step 2 spliceosome<br>GO:0005634 : nucleus<br>GO:0071011 : precatalytic spliceosome<br>GO:0005681 : spliceosomal complex	GO:0003729 : mRNA binding<br>GO:0000166 : nucleotide binding<br>GO:0008187 : poly-pyrimidine tract binding
CG4792	*Dcr-1*	GO:0048813 : dendrite morphogenesis<br>GO:0033227 : dsRNA transport<br>GO:0035195 : gene silencing by miRNA<br>GO:0042078 : germ-line stem cell division<br>GO:0045448 : mitotic cell cycle, embryonic<br>GO:0007279 : pole cell formation<br>GO:0031054 : pre-miRNA processing<br>GO:0035196 : production of miRNAs involved in gene silencing by miRNA<br>GO:0030422 : production of siRNA involved in RNA interference<br>GO:0016246 : RNA interference<br>GO:0007367 : segment polarity determination<br>GO:0035087 : siRNA loading onto RISC involved in RNA interference	GO:0005737 : cytoplasm<br>GO:0005634 : nucleus<br>GO:0016442 : RNA-induced silencing complex	GO:0005524 : ATP binding<br>GO:0016443 : bidentate ribonuclease III activity<br>GO:0003725 : double-stranded RNA binding<br>GO:0004386 : helicase activity<br>GO:0005515 : protein binding
CG4878	*eIF3-S9*	GO:0006413 : translational initiation	GO:0005829 : cytosol<br>GO:0005852 : eukaryotic translation initiation factor 3 complex	GO:0003729 : mRNA binding<br>GO:0000166 : nucleotide binding<br>GO:0003743 : translation initiation factor activity
CG4886	*cyp33*	GO:0000398 : nuclear mRNA splicing, via spliceosome<br>GO:0006911 : phagocytosis, engulfment<br>GO:0006457 : protein folding	GO:0071013 : catalytic step 2 spliceosome<br>GO:0005634 : nucleus<br>GO:0071011 : precatalytic spliceosome	GO:0003729 : mRNA binding<br>GO:0000166 : nucleotide binding<br>GO:0003755 : peptidyl-prolyl cis-trans isomerase activity

				GO:0003723 : RNA binding
CG4887		GO:0000398 : nuclear mRNA splicing, via spliceosome	GO:0071011 : precatalytic spliceosome	GO:0003729 : mRNA binding<br>GO:0003676 : nucleic acid binding<br>GO:0000166 : nucleotide binding<br>GO:0008270 : zinc ion binding
CG4912	*eEF1∂*	GO:0006414 : translational elongation	GO:0005853 : eukaryotic translation elongation factor 1 complex<br>GO:0005875 : microtubule associated complex	GO:0005085 : guanyl-nucleotide exchange factor activity<br>GO:0003746 : translation elongation factor activity
CG4954	*eIF3-S8*	GO:0006413 : translational initiation	GO:0005829 : cytosol<br>GO:0005852 : eukaryotic translation initiation factor 3 complex	GO:0003743 : translation initiation factor activity
CG5168				GO:0046872 : metal ion binding
CG5263	*smg*	GO:0051236 : establishment of RNA localization<br>GO:0017148 : negative regulation of translation<br>GO:0000289 : nuclear-transcribed mRNA poly(A) tail shortening<br>GO:0043488 : regulation of mRNA stability	GO:0005737 : cytoplasm<br>GO:0000932 : cytoplasmic mRNA processing body	GO:0003730 : mRNA 3'-UTR binding<br>GO:0003729 : mRNA binding<br>GO:0005515 : protein binding<br>GO:0030371 : translation repressor activity<br>GO:0000900 : translation repressor activity, nucleic acid binding
CG5439		GO:0007154 : cell communication		GO:0035091 : phosphatidylinositol binding
CG5589				GO:0005524 : ATP binding<br>GO:0004004 : ATP-dependent RNA helicase activity<br>GO:0003729 : mRNA binding
CG5605	*eRF1*	GO:0048102 : autophagic cell death<br>GO:0006605 : protein targeting<br>GO:0035071 : salivary gland cell autophagic cell death<br>GO:0007224 : smoothened signaling pathway<br>GO:0006415 : translational termination	GO:0005737 : cytoplasm<br>GO:0005829 : cytosol<br>GO:0018444 : translation release factor complex	GO:0003747 : translation release factor activity<br>GO:0016149 : translation release factor activity, codon specific<br>GO:0008079 : translation termination factor activity

CG5637	*nos*	GO:0008595 : anterior/posterior axis specification, embryo<br>GO:0001709 : cell fate determination<br>GO:0048813 : dendrite morphogenesis<br>GO:0016321 : female meiosis chromosome segregation<br>GO:0007281 : germ cell development<br>GO:0008354 : germ cell migration<br>GO:0048134 : germ-line cyst formation<br>GO:0042078 : germ-line stem cell division<br>GO:0030718 : germ-line stem cell maintenance<br>GO:0006378 : mRNA polyadenylation<br>GO:0043066 : negative regulation of apoptotic process<br>GO:0045892 : negative regulation of transcription, DNA-dependent<br>GO:0017148 : negative regulation of translation<br>GO:0007314 : oocyte anterior/posterior axis specification<br>GO:0048477 : oogenesis<br>GO:0007280 : pole cell migration<br>GO:0035282 : segmentation<br>GO:0007283 : spermatogenesis	GO:0045495 : pole plasm	GO:0005515 : protein binding<br>GO:0003723 : RNA binding<br>GO:0008270 : zinc ion binding
CG5705		GO:0006415 : translational termination	GO:0005739 : mitochondrion	GO:0016149 : translation release factor activity, codon specific
CG5800		GO:0007526 : larval somatic muscle development<br>GO:0022008 : neurogenesis		GO:0005524 : ATP binding<br>GO:0008026 : ATP-dependent helicase activity<br>GO:0003676 : nucleic acid binding<br>GO:0003724 : RNA helicase activity
CG6341	*Ef1ß*	GO:0006414 : translational elongation	GO:0005829 : cytosol<br>GO:0005853 : eukaryotic translation elongation factor 1 complex<br>GO:0005811 : lipid particle	GO:0005085 : guanyl-nucleotide exchange factor activity<br>GO:0003746 : translation elongation factor activity<br>GO:0003747 : translation release factor activity
CG6375	*pit*		GO:0005730 : nucleolus<br>GO:0005634 : nucleus	GO:0005524 : ATP binding<br>GO:0004004 : ATP-dependent RNA helicase activity

				GO:0003724 : RNA helicase activity<br>GO:0003676 : nucleic acid binding
CG6412	*mtEF-Ts*	GO:0006414 : translational elongation	GO:0005739 : mitochondrion	GO:0003746 : translation elongation factor activity
CG6418				GO:0005524 : ATP binding<br>GO:0004004 : ATP-dependent RNA helicase activity<br>GO:0004386 : helicase activity<br>GO:0003676 : nucleic acid binding
CG6493	*Dcr-2*	GO:0006342 : chromatin silencing<br>GO:0051607 : defense response to virus<br>GO:0009597 : detection of virus<br>GO:0033227 : dsRNA transport<br>GO:0070868 : heterochromatin organization involved in chromatin silencing<br>GO:0007626 : locomotory behavior<br>GO:0006379 : mRNA cleavage<br>GO:0031054 : pre-miRNA processing<br>GO:0030422 : production of siRNA involved in RNA interference<br>GO:0016246 : RNA interference<br>GO:0035087 : siRNA loading onto RISC involved in RNA interference<br>GO:0030423 : targeting of mRNA for destruction involved in RNA interference	GO:0005737 : cytoplasm<br>GO:0005634 : nucleus<br>GO:0016442 : RNA-induced silencing complex	GO:0005524 : ATP binding<br>GO:0008026 : ATP-dependent helicase activity<br>GO:0016887 : ATPase activity<br>GO:0003725 : double-stranded RNA binding<br>GO:0004386 : helicase activity<br>GO:0004525 : ribonuclease III activity<br>GO:0035197 : siRNA binding
CG6539	*Gem3*	GO:0007528 : neuromuscular junction development<br>GO:0022618 : ribonucleoprotein complex assembly	GO:0015030 : Cajal body<br>GO:0005737 : cytoplasm<br>GO:0071254 : cytoplasmic U snRNP body	GO:0005524 : ATP binding<br>GO:0004004 : ATP-dependent RNA helicase activity<br>GO:0003676 : nucleic acid binding<br>GO:0003724 : RNA helicase activity
CG6779	*RpS3*	GO:0006281 : DNA repair<br>GO:0007067 : mitosis	GO:0005737 : cytoplasm<br>GO:0022627 : cytosolic small ribosomal subunit	GO:0003906 : DNA-(apurinic or apyrimidinic site) lyase activity<br>GO:0008534 : oxidized purine base lesion DNA N-glycosylase activity

		GO:0043524 : negative regulation of neuron apoptotic process<br>GO:0006974 : response to DNA damage stimulus<br>GO:0006412 : translation	GO:0005811 : lipid particle<br>GO:0005875 : microtubule associated complex<br>GO:0016363 : nuclear matrix<br>GO:0005634 : nucleus<br>GO:0005840 : ribosome	GO:0003723 : RNA binding<br>GO:0003735 : structural constituent of ribosome
CG6866	*loqs*	GO:0007417 : central nervous system development<br>GO:0048132 : female germ-line stem cell division<br>GO:0030718 : germ-line stem cell maintenance<br>GO:0010586 : miRNA metabolic process<br>GO:0031054 : pre-miRNA processing<br>GO:0030422 : production of siRNA involved in RNA interference<br>GO:0070918 : production of small RNA involved in gene silencing by RNA<br>GO:0070920 : regulation of production of small RNA involved in gene silencing by RNA	GO:0005737 : cytoplasm	GO:0003725 : double-stranded RNA binding<br>GO:0005515 : protein binding
CG6946	*glo*	GO:0051276 : chromosome organization<br>GO:0060810 : intracellular mRNA localization involved in pattern specification process<br>GO:0007311 : maternal specification of dorsal/ventral axis, oocyte, germ-line encoded<br>GO:0048477 : oogenesis<br>GO:0045451 : pole plasm oskar mRNA localization<br>GO:0006417 : regulation of translation	GO:0043234 : protein complex	GO:0003729 : mRNA binding<br>GO:0003676 : nucleic acid binding<br>GO:0000166 : nucleotide binding<br>GO:0005515 : protein binding
CG6961				GO:0003729 : mRNA binding<br>GO:0003676 : nucleic acid binding<br>GO:0000166 : nucleotide binding
CG6987	*SF2*	GO:0007095 : mitotic cell cycle G2/M transition DNA damage checkpoint<br>GO:0006376 : mRNA splice site selection	GO:0071013 : catalytic step 2 spliceosome<br>GO:0005634 : nucleus	GO:0003729 : mRNA binding<br>GO:0003676 : nucleic acid binding

		GO:0022008 : neurogenesis<br>GO:0000398 : nuclear mRNA splicing, via spliceosome	GO:0071011 : precatalytic spliceosome<br>GO:0005681 : spliceosomal complex	GO:0000166 : nucleotide binding<br>GO:0005515 : protein binding
CG7082	*papi*			GO:0003723 : RNA binding
CG7269	*hel25E*	GO:0007052 : mitotic spindle organization<br>GO:0006406 : mRNA export from nucleus<br>GO:0000398 : nuclear mRNA splicing, via spliceosome<br>GO:0000381 : regulation of alternative nuclear mRNA splicing, via spliceosome	GO:0005875 : microtubule associated complex<br>GO:0005634 : nucleus<br>GO:0005681 : spliceosomal complex	GO:0005524 : ATP binding<br>GO:0004004 : ATP-dependent RNA helicase activity<br>GO:0003676 : nucleic acid binding<br>GO:0003724 : RNA helicase activity
CG7903				GO:0003729 : mRNA binding<br>GO:0003676 : nucleic acid binding<br>GO:0000166 : nucleotide binding
CG8053	*eIF-1a*	GO:0000022 : mitotic spindle elongation<br>GO:0007052 : mitotic spindle organization<br>GO:0006911 : phagocytosis, engulfment<br>GO:0007224 : smoothened signaling pathway<br>GO:0006412 : translation<br>GO:0006413 : translational initiation	GO:0005829 : cytosol<br>GO:0016282 : eukaryotic 43S preinitiation complex	GO:0003743 : translation initiation factor activity
CG8190	*eIF2B-gamma*	GO:0071456 : cellular response to hypoxia<br>GO:0006413 : translational initiation	GO:0005829 : cytosol<br>GO:0005851 : eukaryotic translation initiation factor 2B complex	GO:0016779 : nucleotidyltransferase activity<br>GO:0003743 : translation initiation factor activity
CG8205	*fus*	GO:0007173 : epidermal growth factor receptor signaling pathway		GO:0003729 : mRNA binding<br>GO:0003676 : nucleic acid binding<br>GO:0000166 : nucleotide binding
CG8280	*Ef1α48D*	GO:0008340 : determination of adult lifespan<br>GO:0006412 : translation<br>GO:0006414 : translational elongation	GO:0005737 : cytoplasm<br>GO:0005829 : cytosol<br>GO:0005853 : eukaryotic translation elongation factor 1 complex<br>GO:0005811 : lipid particle<br>GO:0005875 : microtubule associated complex	GO:0005525 : GTP binding<br>GO:0003924 : GTPase activity<br>GO:0003746 : translation elongation factor activity

CG8636	*eIF3-S4*	GO:0007067 : mitosis<br>GO:0000022 : mitotic spindle elongation<br>GO:0007052 : mitotic spindle organization<br>GO:0006413 : translational initiation	GO:0005852 : eukaryotic translation initiation factor 3 complex	GO:0003729 : mRNA binding<br>GO:0003676 : nucleic acid binding<br>GO:0000166 : nucleotide binding<br>GO:0003743 : translation initiation factor activity<br>GO:0008270 : zinc ion binding
CG8730	*drosha*	GO:0022008 : neurogenesis<br>GO:0031053 : primary miRNA processing<br>GO:0016075 : rRNA catabolic process	GO:0005634 : nucleus	GO:0003725 : double-stranded RNA binding<br>GO:0004525 : ribonuclease III activity
CG8882	*eIF3-S2 (Trip1)*	GO:0006412 : translation<br>GO:0006413 : translational initiation	GO:0005829 : cytosol<br>GO:0005852 : eukaryotic translation initiation factor 3 complex<br>GO:0005875 : microtubule associated complex	GO:0003743 : translation initiation factor activity
CG9054	*Ddx1*	GO:0001700 : embryonic development via the syncytial blastoderm<br>GO:0006446 : regulation of translational initiation<br>GO:0042254 : ribosome biogenesis<br>GO:0000245 : spliceosomal complex assembly	GO:0005575 : cellular_component	GO:0005524 : ATP binding<br>GO:0008026 : ATP-dependent helicase activity<br>GO:0004386 : helicase activity<br>GO:0003676 : nucleic acid binding
CG9075	*eIF-4a*	GO:0051297 : centrosome organization<br>GO:0006268 : DNA unwinding involved in replication<br>GO:0009950 : dorsal/ventral axis specification<br>GO:0048132 : female germ-line stem cell division<br>GO:0030718 : germ-line stem cell maintenance<br>GO:0007446 : imaginal disc growth<br>GO:0002168 : instar larval development<br>GO:0007067 : mitosis<br>GO:0000022 : mitotic spindle elongation<br>GO:0007052 : mitotic spindle organization<br>GO:0048477 : oogenesis	GO:0005813 : centrosome<br>GO:0005829 : cytosol<br>GO:0016281 : eukaryotic translation initiation factor 4F complex<br>GO:0005875 : microtubule associated complex<br>GO:0072686 : mitotic spindle<br>GO:0005634 : nucleus	GO:0005524 : ATP binding<br>GO:0008026 : ATP-dependent helicase activity<br>GO:0003729 : mRNA binding<br>GO:0000339 : RNA cap binding<br>GO:0003724 : RNA helicase activity<br>GO:0017116 : single-stranded DNA-dependent ATP-dependent DNA helicase activity<br>GO:0003743 : translation initiation factor activity

		GO:0000381 : regulation of alternative nuclear mRNA splicing, via spliceosome<br>GO:0006974 : response to DNA damage stimulus<br>GO:0006413 : translational initiation		
CG9107				GO:0000166 : nucleotide binding
CG9124	*eIF-3p40*	GO:0022008 : neurogenesis<br>GO:0006413 : translational initiation	GO:0005829 : cytosol<br>GO:0005852 : eukaryotic translation initiation factor 3 complex	GO:0003743 : translation initiation factor activity
CG9218	*sm*	GO:0008343 : adult feeding behavior<br>GO:0007411 : axon guidance	GO:0030529 : ribonucleoprotein complex	GO:0003729 : mRNA binding<br>GO:0003676 : nucleic acid binding<br>GO:0000166 : nucleotide binding
CG9373	*rump*	GO:0008595 : anterior/posterior axis specification, embryo<br>GO:0009952 : anterior/posterior pattern specification<br>GO:0008298 : intracellular mRNA localization<br>GO:0007067 : mitosis<br>GO:0007277 : pole cell development<br>GO:0045451 : pole plasm *oskar* mRNA localization<br>GO:0035282 : segmentation	GO:0005737 : cytoplasm<br>GO:0005634 : nucleus<br>GO:0030529 : ribonucleoprotein complex	GO:0003730 : mRNA 3'-UTR binding<br>GO:0003729 : mRNA binding<br>GO:0000166 : nucleotide binding
CG9412	*rin*	GO:0001752 : compound eye photoreceptor fate commitment<br>GO:0000398 : nuclear mRNA splicing, via spliceosome<br>GO:0016318 : ommatidial rotation<br>GO:0007265 : Ras protein signal transduction<br>GO:0006810 : transport	GO:0071013 : catalytic step 2 spliceosome<br>GO:0005829 : cytosol<br>GO:0005811 : lipid particle<br>GO:0071011 : precatalytic spliceosome	GO:0003729 : mRNA binding<br>GO:0003676 : nucleic acid binding<br>GO:0000166 : nucleotide binding<br>GO:0017124 : SH3 domain binding
CG9680	*Dbp73D*	GO:0022008 : neurogenesis		GO:0005524 : ATP binding<br>GO:0008026 : ATP-dependent helicase activity<br>GO:0004004 : ATP-dependent RNA helicase activity
CG9755	*pum*	GO:0008595 : anterior/posterior axis specification, embryo<br>GO:0001709 : cell fate determination	GO:0005737 : cytoplasm	GO:0003730 : mRNA 3'-UTR binding<br>GO:0003723 : RNA binding

		GO:0016477 : cell migration<br>GO:0048813 : dendrite morphogenesis<br>GO:0007281 : germ cell development<br>GO:0042078 : germ-line stem cell division<br>GO:0008258 : head involution<br>GO:0007616 : long-term memory<br>GO:0007067 : mitosis<br>GO:0045786 : negative regulation of cell cycle<br>GO:0042059 : negative regulation of epidermal growth factor receptor signaling pathway<br>GO:0045892 : negative regulation of transcription, DNA-dependent<br>GO:0017148 : negative regulation of translation<br>GO:0000288 : nuclear-transcribed mRNA catabolic process, deadenylation-dependent decay<br>GO:0048477 : oogenesis<br>GO:0007280 : pole cell migration<br>GO:0045727 : positive regulation of translation<br>GO:0008582 : regulation of synaptic growth at neuromuscular junction<br>GO:0050804 : regulation of synaptic transmission<br>GO:0007268 : synaptic transmission		GO:0000900 : translation repressor activity, nucleic acid binding
CG9769	*eIF3-S5*	GO:0048102 : autophagic cell death<br>GO:0035071 : salivary gland cell autophagic cell death<br>GO:0006413 : translational initiation	GO:0005852 : eukaryotic translation initiation factor 3 complex	GO:0003743 : translation initiation factor activity
CG9809	*Spargel*	GO:0008286 : insulin receptor signaling pathway<br>GO:0046627 : negative regulation of insulin receptor signaling pathway<br>GO:0010821 : regulation of mitochondrion organization		GO:0003729 : mRNA binding<br>GO:0003676 : nucleic acid binding<br>GO:0000166 : nucleotide binding

		GO:0006355 : regulation of transcription, DNA-dependent		
CG9841	*Efsec*	GO:0006414 : translational elongation<br>GO:0006451 : translational readthrough		GO:0005525 : GTP binding<br>GO:0003924 : GTPase activity<br>GO:0003746 : translation elongation factor activity
CG9946	*eIF-2α*	GO:0001677 : formation of translation initiation ternary complex<br>GO:0000022 : mitotic spindle elongation<br>GO:0007052 : mitotic spindle organization<br>GO:0006413 : translational initiation	GO:0005829 : cytosol<br>GO:0005850 : eukaryotic translation initiation factor 2 complex<br>GO:0005851 : eukaryotic translation initiation factor 2B complex<br>GO:0005875 : microtubule associated complex	GO:0005525 : GTP binding<br>GO:0003743 : translation initiation factor activity<br>GO:0000049 : tRNA binding
CG10084	*swm*	GO:0006397 : mRNA processing<br>GO:0045879 : negative regulation of smoothened signaling pathway<br>GO:0007346 : regulation of mitotic cell cycle	GO:0005634 : nucleus	GO:0003676 : nucleic acid binding<br>GO:0000166 : nucleotide binding<br>GO:0008270 : zinc ion binding
CG10124	*eIF4E-4*	GO:0006413 : translational initiation	GO:0016281 : eukaryotic translation initiation factor 4F complex	GO:0031370 : eukaryotic initiation factor 4G binding<br>GO:0005515 : protein binding<br>GO:0000340 : RNA 7-methylguanosine cap binding<br>GO:0000339 : RNA cap binding<br>GO:0003743 : translation initiation factor activity
CG10203	*x16*	GO:0006376 : mRNA splice site selection<br>GO:0007399 : nervous system development<br>GO:0000398 : nuclear mRNA splicing, via spliceosome<br>GO:0008380 : RNA splicing	GO:0071013 : catalytic step 2 spliceosome<br>GO:0005634 : nucleus<br>GO:0071011 : precatalytic spliceosome<br>GO:0005681 : spliceosomal complex	GO:0003729 : mRNA binding<br>GO:0003676 : nucleic acid binding<br>GO:0000166 : nucleotide binding<br>GO:0005515 : protein binding<br>GO:0003723 : RNA binding<br>GO:0008270 : zinc ion binding
CG10315	*eIF2B-∂*	GO:0022008 : neurogenesis<br>GO:0006413 : translational initiation	GO:0005829 : cytosol<br>GO:0005851 : eukaryotic translation initiation factor 2B complex	GO:0003743 : translation initiation factor activity
CG10466		GO:0000398 : nuclear mRNA splicing, via spliceosome	GO:0071013 : catalytic step 2 spliceosome	GO:0003729 : mRNA binding

			GO:0071011 : precatalytic spliceosome	GO:0003676 : nucleic acid binding<br>GO:0000166 : nucleotide binding
CG10719	*brat*	GO:0008356 : asymmetric cell division<br>GO:0055060 : asymmetric neuroblast division resulting in ganglion mother cell formation<br>GO:0007411 : axon guidance<br>GO:0007420 : brain development<br>GO:0007402 : ganglion mother cell fate determination<br>GO:0008285 : negative regulation of cell proliferation<br>GO:0007406 : negative regulation of neuroblast proliferation<br>GO:0007405 : neuroblast proliferation<br>GO:0022008 : neurogenesis<br>GO:0030182 : neuron differentiation<br>GO:0048477 : oogenesis<br>GO:0006911 : phagocytosis, engulfment<br>GO:0051726 : regulation of cell cycle<br>GO:0006417 : regulation of translation<br>GO:0016072 : rRNA metabolic process<br>GO:0009303 : rRNA transcription<br>GO:0035282 : segmentation	GO:0035282 : segmentation<br>GO:0045180 : basal cortex<br>GO:0005737 : cytoplasm	GO:0005515 : protein binding<br>GO:0045182 : translation regulator activity<br>GO:0030371 : translation repressor activity<br>GO:0008270 : zinc ion binding
CG10777				GO:0005524 : ATP binding<br>GO:0008026 : ATP-dependent helicase activity<br>GO:0003676 : nucleic acid binding<br>GO:0003724 : RNA helicase activity
CG10868	*orb*	GO:0000578 : embryonic axis specification<br>GO:0007281 : germ cell development<br>GO:0048134 : germ-line cyst formation<br>GO:0007294 : germarium-derived oocyte fate determination<br>GO:0008298 : intracellular mRNA localization<br>GO:0006378 : mRNA polyadenylation<br>GO:0007314 : oocyte anterior/posterior axis specification	GO:0005737 : cytoplasm	GO:0003729 : mRNA binding<br>GO:0000166 : nucleotide binding<br>GO:0005515 : protein binding<br>GO:0017131 : uridine-rich cytoplasmic polyadenylylation element binding

		GO:0007309 : oocyte axis specification<br>GO:0007310 : oocyte dorsal/ventral axis specification<br>GO:0048477 : oogenesis<br>GO:0007315 : pole plasm assembly<br>GO:0045451 : pole plasm oskar mRNA localization<br>GO:0046012 : positive regulation of oskar mRNA translation<br>GO:0045727 : positive regulation of translation<br>GO:0046011 : regulation of oskar mRNA translation		
CG10881	*eIF3-S4*	GO:0006413 : translational initiation	GO:0005852 : eukaryotic translation initiation factor 3 complex	GO:0003729 : mRNA binding<br>GO:0003676 : nucleic acid binding<br>GO:0000166 : nucleotide binding<br>GO:0003743 : translation initiation factor activity<br>GO:0008270 : zinc ion binding
CG10901	*osk*	GO:0008595 : anterior/posterior axis specification, embryo<br>GO:0007281 : germ cell development<br>GO:0007616 : long-term memory<br>GO:0046594 : maintenance of pole plasm mRNA location<br>GO:0007314 : oocyte anterior/posterior axis specification<br>GO:0008103 : oocyte microtubule cytoskeleton polarization<br>GO:0048477 : oogenesis<br>GO:0030719 : P granule organization<br>GO:0007277 : pole cell development<br>GO:0007278 : pole cell fate determination<br>GO:0007279 : pole cell formation<br>GO:0007315 : pole plasm assembly<br>GO:0019094 : pole plasm mRNA localization<br>GO:0007318 : pole plasm protein localization<br>GO:0045727 : positive regulation of translation	GO:0043186 : P granule<br>GO:0045495 : pole plasm	GO:0016787 : hydrolase activity

		GO:0007359 : posterior abdomen determination<br>GO:0043488 : regulation of mRNA stability<br>GO:0046011 : regulation of oskar mRNA translation<br>GO:0007632 : visual behavior<br>GO:0008542 : visual learning		
CG11266		GO:0000398 : nuclear mRNA splicing, via spliceosome<br>GO:0000381 : regulation of alternative nuclear mRNA splicing, via spliceosome<br>GO:0008380 : RNA splicing	GO:0071013 : catalytic step 2 spliceosome<br>GO:0005634 : nucleus<br>GO:0071011 : precatalytic spliceosome<br>GO:0005681 : spliceosomal complex	GO:0003729 : mRNA binding<br>GO:0000166 : nucleotide binding<br>GO:0003723 : RNA binding
CG11334		GO:0006412 : translation<br>GO:0035220 : wing disc development	GO:0005851 : eukaryotic translation initiation factor 2B complex	GO:0045182 : translation regulator activity
CG11454				GO:0003729 : mRNA binding<br>GO:0003676 : nucleic acid binding<br>GO:0000166 : nucleotide binding
CG11505		GO:0008150 : biological_process	GO:0005575 : cellular_component	GO:0003676 : nucleic acid binding
CG11726				GO:0003729 : mRNA binding<br>GO:0003676 : nucleic acid binding<br>GO:0000166 : nucleotide binding
CG12493			GO:0005634 : nucleus	GO:0003725 : double-stranded RNA binding<br>GO:0035197 : siRNA binding
CG13425	*bl*	GO:0035107 : appendage morphogenesis<br>GO:0045165 : cell fate commitment<br>GO:0008283 : cell proliferation<br>GO:0007446 : imaginal disc growth<br>GO:0000381 : regulation of alternative nuclear mRNA splicing, via spliceosome	GO:0000785 : chromatin<br>GO:0005737 : cytoplasm<br>GO:0005634 : nucleus<br>GO:0035062 : omega speckle<br>GO:0030529 : ribonucleoprotein complex	GO:0003729 : mRNA binding<br>GO:0003723 : RNA binding<br>GO:0008134 : transcription factor binding
CG14718			GO:0005622 : intracellular	GO:0003729 : mRNA binding<br>GO:0003676 : nucleic acid binding<br>GO:0000166 : nucleotide binding<br>GO:0008270 : zinc ion binding
CG14891				GO:0003676 : nucleic acid binding<br>GO:0000166 : nucleotide binding

CG16901	*sqd*	GO:0008069 : dorsal/ventral axis specification, ovarian follicular epithelium<br>GO:0009953 : dorsal/ventral pattern formation<br>GO:0007293 : germarium-derived egg chamber formation<br>GO:0008298 : intracellular mRNA localization<br>GO:0006406 : mRNA export from nucleus<br>GO:0033119 : negative regulation of RNA splicing<br>GO:0017148 : negative regulation of translation<br>GO:0000398 : nuclear mRNA splicing, via spliceosome<br>GO:0000184 : nuclear-transcribed mRNA catabolic process, nonsense-mediated decay<br>GO:0007314 : oocyte anterior/posterior axis specification<br>GO:0030720 : oocyte localization involved in germarium-derived egg chamber formation<br>GO:0016325 : oocyte microtubule cytoskeleton organization<br>GO:0048477 : oogenesis<br>GO:0007297 : ovarian follicle cell migration<br>GO:0019094 : pole plasm mRNA localization<br>GO:0045451 : pole plasm oskar mRNA localization<br>GO:0000381 : regulation of alternative nuclear mRNA splicing, via spliceosome<br>GO:0006405 : RNA export from nucleus	GO:0071013 : catalytic step 2 spliceosome<br>GO:0000785 : chromatin<br>GO:0005737 : cytoplasm<br>GO:0005730 : nucleolus<br>GO:0005634 : nucleus<br>GO:0035062 : omega speckle<br>GO:0005703 : polytene chromosome puff<br>GO:0071011 : precatalytic spliceosome<br>GO:0030529 : ribonucleoprotein complex	GO:0003730 : mRNA 3'-UTR binding<br>GO:0003729 : mRNA binding<br>GO:0003676 : nucleic acid binding<br>GO:0000166 : nucleotide binding
CG17492	*mib2*	GO:0046716 : muscle cell homeostasis<br>GO:0007520 : myoblast fusion<br>GO:0016567 : protein ubiquitination	GO:0005737 : cytoplasm<br>GO:0031430 : M band<br>GO:0030018 : Z disc	GO:0032038 : myosin II heavy chain binding<br>GO:0032033 : myosin II light chain binding<br>GO:0004842 : ubiquitin-protein ligase activity<br>GO:0008270 : zinc ion binding
CG17686	*DIP1*		GO:0000785 : chromatin	GO:0003682 : chromatin binding

			GO:0005737 : cytoplasm<br>GO:0005634 : nucleus	GO:0003725 : double-stranded RNA binding<br>GO:0070883 : pre-miRNA binding<br>GO:0005515 : protein binding
CG18259				GO:0003676 : nucleic acid binding<br>GO:0000166 : nucleotide binding
CG31061	*Gr98d*	GO:0050909 : sensory perception of taste	GO:0016021 : integral to membrane	GO:0008527 : taste receptor activity
CG31762	*aret (bruno)*	GO:0007281 : germ cell development<br>GO:0042078 : germ-line stem cell division<br>GO:0030727 : germarium-derived female germ-line cyst formation<br>GO:0002121 : inter-male aggressive behavior<br>GO:0006378 : mRNA polyadenylation<br>GO:0007319 : negative regulation of oskar mRNA translation<br>GO:0017148 : negative regulation of translation<br>GO:0048477 : oogenesis<br>GO:0031536 : positive regulation of exit from mitosis<br>GO:0000381 : regulation of alternative nuclear mRNA splicing, via spliceosome<br>GO:0046011 : regulation of oskar mRNA translation<br>GO:0007286 : spermatid development	GO:0005634 : nucleus<br>GO:0043186 : P granule	GO:0003730 : mRNA 3'-UTR binding<br>GO:0003729 : mRNA binding<br>GO:0000166 : nucleotide binding<br>GO:0005515 : protein binding<br>GO:0003723 : RNA binding
CG32423	*shep*	GO:0042332 : gravitaxis	GO:0005575 : cellular_component	GO:0003729 : mRNA binding<br>GO:0000166 : nucleotide binding<br>GO:0003723 : RNA binding
CG32706		GO:0008150 : biological_process	GO:0005575 : cellular_component	GO:0003676 : nucleic acid binding<br>GO:0000166 : nucleotide binding
CG33100	*4EHP*	GO:0002121 : inter-male aggressive behavior<br>GO:0017148 : negative regulation of translation<br>GO:0022008 : neurogenesis<br>GO:0006413 : translational initiation	GO:0016281 : eukaryotic translation initiation factor 4F complex	GO:0031370 : eukaryotic initiation factor 4G binding<br>GO:0005515 : protein binding<br>GO:0000340 : RNA 7-methylguanosine cap binding<br>GO:0003743 : translation initiation factor activity<br>GO:0030371 : translation repressor activity

CG33106	*mask*	GO:0019730 : antimicrobial humoral response<br>GO:0008283 : cell proliferation<br>GO:0009987 : cellular process<br>GO:0051298 : centrosome duplication<br>GO:0001751 : compound eye photoreceptor cell differentiation<br>GO:0007016 : cytoskeletal anchoring at plasma membrane<br>GO:0007169 : transmembrane receptor protein tyrosine kinase signaling pathway	GO:0005811 : lipid particle	GO:0003723 : RNA binding<br>GO:0005200 : structural constituent of cytoskeleton
CG33197	*mbl*	GO:0006915 : apoptotic process<br>GO:0001751 : compound eye photoreceptor cell differentiation<br>GO:0009790 : embryo development<br>GO:0007517 : muscle organ development<br>GO:0007422 : peripheral nervous system development<br>GO:0045924 : regulation of female receptivity<br>GO:0007525 : somatic muscle development	GO:0005737 : cytoplasm<br>GO:0005634 : nucleus<br>GO:0048471 : perinuclear region of cytoplasm	GO:0003677 : DNA binding<br>GO:0003676 : nucleic acid binding<br>GO:0008270 : zinc ion binding
CG34354				GO:0003729 : mRNA binding<br>GO:0003676 : nucleic acid binding<br>GO:0000166 : nucleotide binding
CG40351	*Set1*	GO:0006338 : chromatin remodeling<br>GO:0051568 : histone H3-K4 methylation<br>GO:0080182 : histone H3-K4 trimethylation	GO:0005634 : nucleus<br>GO:0048188 : Set1C/COMPASS complex	GO:0042800 : histone methyltransferase activity (H3-K4 specific)<br>GO:0018024 : histone-lysine N-methyltransferase activity<br>GO:0003676 : nucleic acid binding<br>GO:0000166 : nucleotide binding
