# Supplementary material for: Extensive Use of RNA-Binding Proteins in Drosophila Sensory Neuron Dendrite Morphogenesis
Source: G3 (Bethesda). 2013 Dec 17;4(2):297–306. doi: 10.1534/g3.113.009795 (PMC3931563; doi:10.1534/g3.113.009795)
Supplement: Supporting Information [file supp_g3.113.009795_TableS1.pdf]

Table S1   List of RNAi stocks screened.

VDRC STOCK NUMBERS

10192	100563	103141	104978	106257
13305	100611	103355	105054	106318
17003	100693	103365	105107	106321
17065	100702	103411	105121	106393
20321	100709	103427	105135	106475
21379	100722	103665	105148	106636
21763	100723	103704	105254	106645
22837	100732	103708	105271	106696
22846	100739	103728	105291	106734
23677	100775	103735	105322	106754
24725	100805	103751	105325	106762
24889	100813	103769	105437	106833
26045	100817	103789	105486	106944
26243	101060	103791	105495	106972
26472	101288	103913	105500	106994
27752	101399	103940	105543	107004
27776	101412	103972	105585	107007
28072	101435	104096	105612	107013
29116	101465	104156	105619	107031
31324	101508	104160	105672	107063
31364	101513	104183	105704	107064
34210	101537	104187	105763	107112
34711	101555	104253	105825	107147
35288	101567	104327	105883	107153
37863	101740	104334	105907	107268
40352	101765	104342	105907	107282
40683	101765	104351	105949	107304
44895	101781	104379	105950	107385
45027	101925	104401	105954	107445
47973	102118	104403	105963	107459
49506	102159	104471	105991	107575
50094	102173	104481	105992	107593
100001	102360	104502	106041	107595
100226	102360	104562	106047	107709
100291	102442	104715	106078	107723
100310	102597	104865	106079	107750
100356	102736	104876	106189	107829
100546	102825	104941	106240	107953

107958	108216	108900	109689	110143
107993	108310	108916	109739	110165
107993	108351	108933	109742	110357
107999	108358	108947	109762	110410
108026	108376	108950	109762	110441
108065	108552	108993	109782	110451
108072	108580	109212	109796	110452
108072	108642	109221	109892	110457
108083	108653	109436	109911	110472
108094	108666	109465	109951	110476
108158	108734	109500	110008	110514
108169	108828	109602	110075	110518
108186	108847	109672	110102	

NIG STOCK NUMBERS

1316R-1	4886R-1	7185R-2	11454R-4	17697R-1
2931R-3	4887R-3	7697R-1	11505R-2	17838R
3056R-1	5263R-2	7879R-1	11726R-1	18179
3335R-1	5439R-1	7903R-1	14035R-1	18259R-3
4035R-1	5808R-1	8933	14230R-1	20839
4119R-2	6946R-1	9346R-1	14641R-1	27752
4262R-4	6961R-3	10868R-1	14891R-2	32169R-2
4824R-5	6995R-2	10948R-3	14900R-1	45815
4878R-3	6999R-3	11266R-3	17540-R	49473

TRiP (Bloomington) STOCK NUMBERS

25970	28036	30518
26293	28049	34646
26745	28071	
27040	28314	
27051	28360	
27320	28371	
27484	28566	
27565	28590	
27659	28931	
27704	28991	

OTHER

UAS-nosRNAi (Menon et al. 2002)

UAS-pumRNAi (Menon et al. 2002)
