# Supplementary material for: Extensive Use of RNA-Binding Proteins in Drosophila Sensory Neuron Dendrite Morphogenesis
Source: G3 (Bethesda). 2013 Dec 17;4(2):297–306. doi: 10.1534/g3.113.009795 (PMC3931563; doi:10.1534/g3.113.009795)
Supplement: Supporting Information [file supp_g3.113.009795_TableS2.pdf]

Table S2 Homologs of *Drosophila* RBPs and translational factors with functions in dendrite morphogenesis

***Drosophila* CG**	***Drosophila* Protein Name**	**BLAST - Fly to Human**	**Human Homolog**	**BLAST - Fly to Mouse***	**Mouse Homolog**	**BLAST - Fly to *C. elegans***	***C. elegans* Homolog**
CG1873	Elongation factor 1 alpha 100E (EF1 alpha 100E)	0	elongation factor 1-alpha 1 (NP_001393)	0	elongation factor 1-alpha 1 (NP_034236)	0	EEF-1A.2 (R03G5.1a)/EEF-1A.1 (F31E3.5)
CG3056	Sister-of-Sex-lethal (Ssx)	6e-53	ELAV-like protein 4 (NP_001138246)	3e-53	ELAV-like protein 4 (NP_001156869)	1e-47	EXC-7 (F35H8.5)
CG3249	Spoonbill (Spoon)/yu	1e-46	A-kinase anchor protein 1 (NP_003479)	3e-46	A-kinase anchor protein 1 (NP_001036006)	3e-30	C56G2.1
CG3582	U2 small nuclear riboprotein auxiliary factor 38 (U2AF38)	3e-119	splicing factor U2AF 35 kDa subunit (NP_001020374)	3e-119	splicing factor U2AF 35 kDa subunit (NP_001157241)	9e-90	UAF-2 (Y116A8C.35)
CG3806	eIF2B-ε	2e-69	translation initiation factor eIF-2B subunit epsilon (NP_003898)	5e-98	translation initiation factor eIF-2B subunit epsilon (NP_758469)	3e-22	D2085.3
CG4119		2e-47	RNA-binding protein 25 (NP_067062)	2e-47	RNA-binding protein 25 (NP_081625)	8e-28	W04D2.6
CG4152	Lethal(2)35Df (L(2)35Df)	0	superkiller viralicidic activity 2-like 2 (NP_056175)	0	superkiller viralicidic activity 2-like 2 (NP_082427)	0	MTR-4 (W08D2.7)
CG4153	Eukaryotic initiation factor 2β (eIF-2β)	4e-101	eukaryotic translation initiation factor 2 subunit 2 (NP_003899)	2e-102	eukaryotic translation initiation factor 2 subunit 2 (NP_080306)	1e-72	ITFB-1 (K04G2.1)
CG4262	Embryonic lethal abnormal vision (Elav)	9e-126	ELAV-like protein 4 (NP_001138246	7e-126	ELAV-like protein 4 (NP_001156869)	3e-75	EXC-7 (F35H8.5)
CG4396	Found in neurons (Fne)	2e-146	ELAV-like protein 2 (NP_001164666)	7e-147	ELAV-like protein 4 (NP_001156869)	4e-85	EXC-7 (F35H8.5)
CG4528	Sans fille (Snf)	3e-103	U2 small nuclear ribonucleoprotein B (NP_003083)	1e-101	U2 small nuclear ribonucleoprotein B (NP_067310)	5e-70	RNP-3 (K08D10.3)
CG4602	Srp54	2e-55	splicing regulatory glutamine/lysine-rich protein 1 (NP_001070667)	7e-30	splicing factor, arginine/serine-rich 11 (NP_001087221)	8e-45	RSP-7 (D2089.1)

CG4792	Dicer-1 (Dcr-1)	3e-122	endoribonuclease Dicer (NP_001182502)	2e-123	endoribonuclease Dicer (NP_683750)	3e-124	DCR-1 (K12H4.8)
CG4878	eIF3-S9	0	eukaryotic translation initiation factor 3 subunit B (NP_003742)	0	eukaryotic translation initiation factor 3 subunit B (NP_598677)	6e-114	EIF-3.b (Y54E2A.11)
CG4886	Cyclophilin-33 (Cyp33)	9e-146	peptidyl-prolyl cis-trans isomerase E (NP_006103)	2e-144	peptidyl-prolyl cis-trans isomerase E (NP_062362)	5e-124	CYN-13 (Y116A8C.34)
CG4887		4e-98	RNA-binding protein 10 (NP_001191397)	2e-99	RNA-binding protein 10 (NP_663602)	2e-23	T08B2.5
CG4912	eEF1δ	2e-67	elongation factor 1-delta (NP_001951)	4e-66	elongation factor 1-delta (NP_075729)	6e-40	EEF-1b.1 (F54H12.6)
CG4954	eIF3-S8	0	eukaryotic translation initiation factor 3 subunit C (NP_003743)	0	Eukaryotic translation initiation factor 3, subunit C (NP_666312)	0	EIF-3.c (T23D8.4)
CG5168		6e-163	WD repeat and FYVE domain-containing protein 2 (NP_443182)	8e-163	WD repeat and FYVE domain-containing protein 2 (NP_780755)	2e-98	WDFY-2 (D2013.2)
CG5263	Smaug (Smg)	9e-24	Smaug homolog 2 (NP_060498)	3e-24	smaug homolog 1 (NP_001156905)	7e-17	ZC190.4
CG5439		5e-30	sorting nexin-29 (NP_115543)	4e-29	sorting nexin-29 (NP_083240)	2e-08	F13E9.1
CG5589		8e-171	probable ATP-dependent RNA helicase DDX52 (NP_008941)	5e-60	probable ATP-dependent RNA helicase DDX23 (NP_001074450)	6e-112	R05D11.4
CG5605	Eukaryotic release factor 1 (eRF1)	0	eukaryotic peptide chain release factor subunit 1 (NP_004721)	0	eukaryotic peptide chain release factor subunit 1 (NP_659115)	0	T05H4.6
CG5637	Nanos (Nos)	1e-18	nanos homolog 1 (NP_9556319)	5e-19	nanos homolog 1 (NP_848508)	1e-07	NOS-1 (R03D7.7)
CG5705		4e-90	peptide chain release factor 1-like, mitochondrial isoform a (NP_061914)	6e-94	peptide chain release factor 1-like, mitochondrial precursor (NP_780583)	1e-67	W03F8.3
CG5800		0	probable ATP-dependent RNA	0	probable ATP-dependent RNA	1e-172	Y23H5B.6

			helicase DDX10 (NP_004389)		helicase DDX10 (NP_084212)		
CG6341	Elongation factor 1 β (Ef1β)	3e-78	elongation factor 1-beta (NP_001950)	8e-84	elongation factor 1-beta (NP_061266)	5e-63	EEF-1b.1 (F54H12.6)
CG6375	Pitchoune (Pit)	0	ATP-dependent RNA helicase DDX18 (NP_006764)	0	ATP-dependent RNA helicase DDX18 (NP_080136)	1e-175	B0511.6
CG6412	mtEF-Ts	6e-55	elongation factor Ts, mitochondrial isoform 2 precursor (NP_005717.3)	7e-60	elongation factor Ts, mitochondrial precursor (NP_079813)	9e-52	TSFM-1 (F55C5.5)
CG6418		0	ATP-dependent RNA helicase DDX42 (NP_031398)	0	ATP-dependent RNA helicase DDX42 (NP_082350)	0	C46F11.4
CG6493	Dicer-2 (Dcr-2)	6e-101	endoribonuclease Dicer (NP_001182502)	3e-103	endoribonuclease Dicer (NP_683750)	3e-70	DCR-1 (K12H4.8)
CG6539	Gemin3 (Gem3)	3e-77	probable ATP-dependent RNA helicase DDX20 (NP_009135)	7e-78	probable ATP-dependent RNA helicase DDX20 (NP_059093)	2e-67	CGH-1 (C07H6.5)
CG6779	Ribosomal protein S3 (RpS3)	1e-139	40S ribosomal protein S3 (NP_000996)	6e-140	40S ribosomal protein S3 (NP_036182)	6e-124	RPS-3 (C23G10.3)
CG6866	Loquacious (Loqs)	2e-49	interferon-inducible double stranded RNA-dependent protein kinase activator A (NP_001132990)	9e-50	interferon-inducible double stranded RNA-dependent protein kinase activator A (NP_036001)	6e-09	D1037.1
CG6946	Glorund (Glo)	9e-50	heterogeneous nuclear ribonucleoprotein F (NP_004957)	1e-49	heterogeneous nuclear ribonucleoprotein F (NP_598595)	3e-32	SYM-2 (ZK1067.6)
CG6961		6e-13	polymerase delta-interacting protein 3 (NP_835237)	1e-12	polymerase delta interacting protein 3 (NP_848742)	No Hit	No Hit
CG6987	SF2	1e-81	serine/arginine-rich splicing factor 1 (NP_008855)	3e-76	serine/arginine-rich splicing factor 1 (NP_001071635)	1e-70	RSP-3 (Y111B2A.18)
CG7082	Papi	7e-53	tudor and KH domain-containing protein (NP_006853)	2e-51	tudor and KH domain-containing protein (NP_082583)	1e-42	C56G2.1

CG7269	Helicase at 25E (Hel25E)	0	spliceosome RNA helicase DDX39B (NP_004631)	0	spliceosome RNA helicase Ddx39b (NP_062667)	0	HEL-1 (C26D10.2)
CG7903		2e-15	RNA-binding protein 14 (NP_006319)	7e-15	RNA-binding protein 14 (NP_063922)	No Hit	No Hit
CG8053	Eukaryotic inititation factor 1A (eIF-1A)	3e-75	eukaryotic translation initiation factor 1A, X-chromosomal (NP_001403)	1e-74	eukaryotic translation initiation factor 1A (NP_034250)	2e-63	EIF-1.a (H06H21.3)
CG8190	eIF2Bgamma	3e-86	translation initiation factor eIF-2B subunit gamma (NP_065098)	2e-91	eukaryotic translation initiation factor 2B, subunit 3 gamma (NP_001104747)	1e-39	PPP-1 (C15F1.4)
CG8205	Fusilli (Fus)	6e-150	epithelial splicing regulatory protein 1 (NP_001116297)	7e-150	epithelial splicing regulatory protein 1 (NP_918944)	5e-82	SYM-2 (ZK1067.6)
CG8280	Elongation factor 1 alpha 48D (EF1 alpha 48D)	0	elongation factor 1-alpha 1 (NP_001393)	0	elongation factor 1-alpha 1 (NP_034236)	0	EEF-1A.2 (R03G5.1a)/EEF-1A.1 (F31E3.5)
CG8636	eIF3-S4	1e-70	eukaryotic translation initiation factor 3 subunit G (NP_003746)	1e-70	eukaryotic translation initiation factor 3 subunit G (NP_058572)	4e-44	EIF-3.g (F22B5.2)
CG8730	Drosha	0	ribonuclease 3 (NP_001093882)	0	ribonuclease 3 (NP_081075)	0	DRSH-1 (F26E4.10)
CG8882	Trip1/eIF3-S2	2e-149	eukaryotic translation initiation factor 3 subunit I (NP_003748)	5e-149	eukaryotic translation initiation factor 3 subunit I (NP_061269)	4e-109	EIF-3.i (Y74C10AR.1)
CG9054	Dead-box-1 (Ddx1)	0	ATP-dependent RNA helicase DDX1 (NP_004930)	0	ATP-dependent RNA helicase DDX1 (NP_598801)	0	Y55F3BR.1
CG9075	Eukaryotic initiation factor 4a (eIF-4a)	0	eukaryotic initiation factor 4A-II (NP_001958)	0	eukaryotic initiation factor 4A-III (NP_619610)	0	INF-1 (F57B9.6)
CG9107		4e-48	ribosomal RNA-processing protein 7 homolog A (NP_056518)	6e-51	ribosomal RNA-processing protein 7 homolog A (NP_083377)	1e-11	ZC434.3
CG9124	Eukaryotic initiation factor 3 p40 (eIF-3p40)	3e-99	eukaryotic translation initiation factor 3 subunit H (NP_003747)	1e-100	eukaryotic translation initiation factor 3 subunit H (NP_542366)	1e-49	EIF-3.h (C41D11.2)

CG9218	Smooth (Sm)	2e-100	Heterogeneous nuclear ribonucleoprotein L (NP_001005335)	3e-99	Heterogeneous nuclear ribonucleoprotein L (NP_796275)	3e-69	C44B7.2
CG9373	Rumpelstiltskin (Rump)	2e-25	Myelin expression factor 2 (NP_034982.2)	9e-47	Myelin expression factor 2 (NP_057216.2)	1e-46	C25A1.4
CG9412	Rasputin (Rin)	3e-47	ras GTPase-activating protein-binding protein 2 (NP_987100)	4e-47	ras GTPase-activating protein-binding protein 2 (NP_035946)	3e-10	K08F4.2
CG9680	Dead box protein 73D (Dbp73D)	2e-107	ATP-dependent RNA helicase DDX51 (NP_778236)	8e-106	ATP-dependent RNA helicase DDX51 (NP_081432)	4e-59	ZK686.2
CG9755	Pumilio (Pum)	0	pumilio homolog 2 (NP_056132)	0	Pumilio homolog 2 (NP_001153694)	6e-128	PUF-9 (W06B11.2)
CG9769	eIF3-S5	3e-94	eukaryotic translation initiation factor 3 subunit F (NP_003745)	4e-93	eukaryotic translation initiation factor 3 subunit F (NP_079620)	4e-47	EIF-3.f (D2013.7)
CG9809	Spargel	3e-24	peroxisome proliferator-activated receptor gamma coactivator-related protein 1 (NP_055877)	2e-24	peroxisome proliferator-activated receptor gamma coactivator-related protein 1 (NP_001074683)	2e-05	RPN-7 (K04G7.10)
CG9841	EfSec	1e-146	selenocysteine-specific elongation factor (NP_068756)	7e-151	selenocysteine-specific elongation factor (NP_075547)	2e-68	SELB-1 (C47B2.7)
CG9946	Eukaryotic translation initiation factor 2α (eIF- 2α)	4e-134	eukaryotic translation initiation factor 2 subunit 1 (NP_004085)	2e-134	eukaryotic translation initiation factor 2 subunit 1 (NP_080390)	2e-108	Y37E3.10
CG10084	Second mitotic wave missing (Swm)	4e-51	RNA-binding protein 26 (NP_071401)	5e-52	RNA-binding protein 26 (NP_598838)	3e-16	B0336.3
CG10124	eIF4E-4	6e-63	Eukaryotic translation initiation factor 4E (NP_001959)	2e-63	eukaryotic translation initiation factor 4E (NP_031943)	2e-54	IFE-3 (B0348.6)
CG10203	X16	7e-35	serine/arginine-rich splicing factor 3 (NP_003008)	2e-34	serine/arginine-rich splicing factor 7 (NP_001026854)	2e-25	RSP-6 (C33H5.12b)

CG10315	eIF2B-δ	3e-123	translation initiation factor eIF-2B subunit delta (NP_001029288)	1e-123	translation initiation factor eIF-2B subunit delta (NP_034252)	5e-22	F11A3.2
CG10466		7e-66	RNA-binding motif protein, X-linked 2 (NP_057108)	3e-65	RNA-binding motif protein, X-linked 2 (NP_775552)	2e-48	C30B5.4
CG10719	Brain tumor (Brat)	3e-42	brain expressed ring finger; tripartite motif-containing protein 3 (NP_001234936)	5e-42	tripartite motif-containing protein 3 (NP_061368)	2e-172	NCL-1 (ZK112.2)
CG10777		0	probable ATP-dependent RNA helicase DDX5 (NP_004387)	0	probable ATP-dependent RNA helicase DDX5 (NP_031866)	3e-167	F58E10.3
CG10868	Oo18 RNA binding protein (Orb)	2e-126	cytoplasmic polyadenylation element-binding protein 1 (NP_001073002)	2e-124	cytoplasmic polyadenylation element-binding protein 1 (NP_001239455)	8e-69	CPB-3 (B0414.5)
CG10881	eIF3-SG2	1e-73	eukaryotic translation initiation factor 3 subunit G (NP_003746)	6e-74	eukaryotic translation initiation factor 3 subunit G (NP_058572)	3e-43	EIF-3.g (F22B5.2)
CG10901	Oskar (Osk)	No Hit	No Hit	No Hit	No Hit	No Hit	No Hit
CG11266		8e-126	RNA-binding protein 39 (NP_001229529)	7e-124	RNA-binding protein 39 (NP_573505)	6e-84	Y55F3AM.3
CG11334		2e-142	methylthioribose-1-phosphate isomerase (NP_001026897)	4e-129	methylthioribose-1-phosphate isomerase (NP_080699)	5e-69	C01G10.9
CG11454		6e-23	RNA-binding protein 7 (NP_057174)	4e-22	RNA-binding protein 7 (NP_659197)	5e-10	SAP-49 (C08B11.5)
CG11505		3e-71	La-related protein 4 (NP_001164275)	2e-70	La-related protein 4 (NP_001074417)	1e-33	LARP-5 (T12F5.5)
CG11726		7e-10	eukaryotic translation initiation factor 4H (NP_071496)	7e-10	eukaryotic translation initiation factor 4H (NP_291039)	8e-06	HRPF-1 (W02D3.11)
CG12493		8e-06	interleukin enhancer-binding factor 3 (NP_703194)	3e-06	interleukin enhancer-binding factor 3 (NP_001036172)	No Hit	No Hit
CG13425	Bancal (Bl)	3e-40	Heterogeneous nuclear	4e-40	Heterogeneous nuclear	2e-39	F26B1.2

			ribonucleoprotein K (NP_112553)		ribonucleoprotein K (NP_079555)		
CG14718		2e-21	TATA-binding protein-associated factor 2N (NP_003478)	4e-21	TATA-binding protein-associated factor 2N (NP_081703)	2e-08	FUST-1 (C27H5.3)
CG14891		7e-07	F-box/LRR-repeat protein 13 (NP_659469)	2e-06	F-box/LRR-repeat protein 14 (NP_598701)	No Hit	No Hit
CG16901	Squid (Sqd)	5e-51	Heterogeneous nuclear ribonucleoprotein D0 (NP_002129)	6e-51	Heterogeneous nuclear ribonucleoprotein D0 (NP_031542)	3e-41	SQD-1 (Y73B6BL.6)
CG17492	Mindbomb 2 (Mib2)	0	E3 ubiquitin-protein ligase MIB2 (NP_001164158)	0	E3 ubiquitin-protein ligase MIB2 (NP_001243036)	7e-23	T28D6.4
CG17686	Disco interacting protein 1 (DIP1)	2e-15	double-stranded RNA-specific editase 1 (NP_001103)	2e-15	double-stranded RNA-specific editase 1 (NP_001020008)	No Hit	No Hit
CG18259		4e-13	polymerase delta-interacting protein 3 (NP_835237)	8e-13	polymerase delta interacting protein 3 (NP_848742)	No Hit	No Hit
CG31061	Gustatory receptor 98d (Gr98d)	No Hit	No Hit	No Hit	No Hit	No Hit	No Hit
CG31762	Arrest (Aret)/Bruno (Bru)	4e-131	CUGBP Elav-like family member 2 (NP_001020248)	3e-133	CUGBP Elav-like family member 2 (NP_001103702)	9e-118	ETR-1 (T01D1.2f)
CG32423	Alan shepard (Shep)	2e-81	RNA-binding motif, single-stranded-interacting protein 2 (NP_002889)	2e-82	RNA-binding motif, single-stranded-interacting protein 2 (NP_001034169)	5e-60	SUP-26 (R10E4.2)
CG32706		5e-16	activator of basal transcription 1 (NP_037507)	1e-16	activator of basal transcription 1 (NP_038952)	3e-11	F57B10.8
CG33100	4EHP	8e-44	eukaryotic translation initiation factor 4E type 2 (NP_004837)	1e-44	eukaryotic translation initiation factor 4E type 2 (NP_075803)	2e-36	IFE-4 (C05D9.5)
CG33106	Multiple ankyrin repeats single KH domain (Mask)	0	ankyrin repeat and KH domain-containing protein 1 (NP_060217)	0	ankyrin repeat and KH domain-containing protein 1 (NP_780584)	2e-154	R11A8.7

CG33197	Muscleblind (Mbl)	2e-42	muscleblind-like protein 1 (NP_066368)	1e-42	muscleblind-like protein 1 (NP_064391)	3e-57	MBL-1 (K02H8.1)
CG34354		1e-79	nucleolysin TIA-1 isoform p40 (NP_071505)	6e-79	nucleolysin TIA-1 (NP_035715)	1e-77	TIAR-1 (C18A3.5)
CG40351	Set1	2e-100	histone-lysine N-methyltransferase SETD1A (NP_055527)	8e-100	SET domain containing 1A (NP_821172)	2e-71	SET-2 (C26E6.9)

BLAST-P search of RBPs and translational factors that are required for dendrite morphogenesis in *Drosophila* da neurons to identify the most homologous human, mouse and *C. elegans* proteins. For *Drosophila* proteins that exist as multiple isoforms, the longest isoform was used. E values reported as 0 are less than 1e-180. “No Hit” indicates that there are no homologs with an E value greater than 1e-05.
