# Supplementary material for: Extensive Use of RNA-Binding Proteins in Drosophila Sensory Neuron Dendrite Morphogenesis
Source: G3 (Bethesda). 2013 Dec 17;4(2):297–306. doi: 10.1534/g3.113.009795 (PMC3931563; doi:10.1534/g3.113.009795)
Supplement: Supporting Information [file supp_g3.113.009795_TableS3.pdf]

Table S3 The gene ontology (GO) terms for the RBPs and Translational Factors that are required for dendrite morphogenesis in *Drosophila* da neurons were found by searching The Gene Ontology website (www.geneontology.org database release May 26, 2012). The GO terms are categorized as Biological Process, Cellular Component, and Molecular Function.

CG	Gene Name	Biological Process	Cellular Component	Molecular Function
CG1873	*Ef1α100E*	GO:0022008 : neurogenesis<br>GO:0006412 : translation<br>GO:0006414 : translational elongation	GO:0005737 : cytoplasm<br>GO:0005853 : eukaryotic translation elongation factor 1 complex<br>GO:0005811 : lipid particle	GO:0005525 : GTP binding<br>GO:0003924 : GTPase activity<br>GO:0003746 : translation elongation factor activity
CG3056	*ssx*			GO:0003729 : mRNA binding<br>GO:0000166 : nucleotide binding<br>GO:0003723 : RNA binding
CG3249	*spoon (yu)*	GO:0007298 : border follicle cell migration<br>GO:0060269 : centripetally migrating follicle cell migration<br>GO:0051276 : chromosome organization<br>GO:0046843 : dorsal appendage formation<br>GO:0008069 : dorsal/ventral axis specification, ovarian follicular epithelium<br>GO:0008298 : intracellular mRNA localization<br>GO:0007616 : long-term memory<br>GO:0008070 : maternal determination of dorsal/ventral axis, ovarian follicular epithelium, germ-line encoded<br>GO:0030837 : negative regulation of actin filament polymerization<br>GO:0008104 : protein localization<br>GO:0006403 : RNA localization<br>GO:0006099 : tricarboxylic acid cycle	GO:0005794 : Golgi apparatus<br>GO:0005739 : mitochondrion	GO:0051018 : protein kinase A binding<br>GO:0003723 : RNA binding
CG3582	*U2af38*	GO:0007052 : mitotic spindle organization<br>GO:0000398 : nuclear mRNA splicing, via spliceosome<br>GO:0000381 : regulation of alternative nuclear mRNA splicing, via spliceosome<br>GO:0008380 : RNA splicing	GO:0005634 : nucleus<br>GO:0071011 : precatalytic spliceosome<br>GO:0005681 : spliceosomal complex<br>GO:0005686 : U2 snRNP	GO:0003729 : mRNA binding<br>GO:0000166 : nucleotide binding<br>GO:0008187 : poly-pyrimidine tract binding<br>GO:0008270 : zinc ion binding
CG3806	*eIF2B-ε*	GO:0016070 : RNA metabolic process	GO:0005829 : cytosol	GO:0005085 : guanyl-nucleotide exchange factor activity

		GO:0006413 : translational initiation	GO:0005851 : eukaryotic translation initiation factor 2B complex	GO:0016779 : nucleotidyltransferase activity<br>GO:0003743 : translation initiation factor activity
CG4119		GO:0000398 : nuclear mRNA splicing, via spliceosome<br>GO:0000381 : regulation of alternative nuclear mRNA splicing, via spliceosome	GO:0005634 : nucleus<br>GO:0071011 : precatalytic spliceosome	GO:0003729 : mRNA binding<br>GO:0003676 : nucleic acid binding<br>GO:0000166 : nucleotide binding
CG4152	*l(2)35Df*	GO:0022008 : neurogenesis<br>GO:0000398 : nuclear mRNA splicing, via spliceosome	GO:0071013 : catalytic step 2 spliceosome	GO:0005524 : ATP binding<br>GO:0004004 : ATP-dependent RNA helicase activity<br>GO:0003676 : nucleic acid binding
CG4153	*eIF-2ß*	GO:0016199 : axon midline choice point recognition<br>GO:0001677 : formation of translation initiation ternary complex<br>GO:0000022 : mitotic spindle elongation<br>GO:0007052 : mitotic spindle organization<br>GO:0006413 : translational initiation	GO:0005829 : cytosol<br>GO:0005850 : eukaryotic translation initiation factor 2 complex<br>GO:0005875 : microtubule associated complex	GO:0005525 : GTP binding<br>GO:0003743 : translation initiation factor activity<br>GO:0000049 : tRNA binding
CG4262	*elav*	GO:0007417 : central nervous system development<br>GO:0031441 : negative regulation of mRNA 3'-end processing<br>GO:0007399 : nervous system development<br>GO:0016070 : RNA metabolic process	GO:0015030 : Cajal body<br>GO:0005634 : nucleus	GO:0003729 : mRNA binding<br>GO:0000166 : nucleotide binding<br>GO:0008266 : poly(U) RNA binding<br>GO:0003723 : RNA binding
CG4396	*fne*	GO:0051252 : regulation of RNA metabolic process	GO:0005737 : cytoplasm	GO:0003729 : mRNA binding<br>GO:0000166 : nucleotide binding<br>GO:0008266 : poly(U) RNA binding
CG4528	*snf*	GO:0019099 : female germ-line sex determination<br>GO:0000398 : nuclear mRNA splicing, via spliceosome<br>GO:0048477 : oogenesis<br>GO:0007539 : primary sex determination, soma	GO:0071013 : catalytic step 2 spliceosome<br>GO:0005634 : nucleus<br>GO:0071011 : precatalytic spliceosome<br>GO:0043234 : protein complex	GO:0003729 : mRNA binding<br>GO:0000166 : nucleotide binding<br>GO:0005515 : protein binding<br>GO:0035614 : snRNA stem-loop binding

		GO:0000381 : regulation of alternative nuclear mRNA splicing, via spliceosome<br>GO:0008380 : RNA splicing	GO:0030532 : small nuclear ribonucleoprotein complex<br>GO:0005681 : spliceosomal complex<br>GO:0005685 : U1 snRNP<br>GO:0005692 : U11 snRNP<br>GO:0005686 : U2 snRNP	GO:0030619 : U1 snRNA binding<br>GO:0030620 : U2 snRNA binding
CG4602	*Srp54*	GO:0006376 : mRNA splice site selection<br>GO:0022008 : neurogenesis<br>GO:0000398 : nuclear mRNA splicing, via spliceosome<br>GO:0000381 : regulation of alternative nuclear mRNA splicing, via spliceosome	GO:0071013 : catalytic step 2 spliceosome<br>GO:0005634 : nucleus<br>GO:0071011 : precatalytic spliceosome<br>GO:0005681 : spliceosomal complex	GO:0003729 : mRNA binding<br>GO:0000166 : nucleotide binding<br>GO:0008187 : poly-pyrimidine tract binding
CG4792	*Dcr-1*	GO:0048813 : dendrite morphogenesis<br>GO:0033227 : dsRNA transport<br>GO:0035195 : gene silencing by miRNA<br>GO:0042078 : germ-line stem cell division<br>GO:0045448 : mitotic cell cycle, embryonic<br>GO:0007279 : pole cell formation<br>GO:0031054 : pre-miRNA processing<br>GO:0035196 : production of miRNAs involved in gene silencing by miRNA<br>GO:0030422 : production of siRNA involved in RNA interference<br>GO:0016246 : RNA interference<br>GO:0007367 : segment polarity determination<br>GO:0035087 : siRNA loading onto RISC involved in RNA interference	GO:0005737 : cytoplasm<br>GO:0005634 : nucleus<br>GO:0016442 : RNA-induced silencing complex	GO:0005524 : ATP binding<br>GO:0016443 : bidentate ribonuclease III activity<br>GO:0003725 : double-stranded RNA binding<br>GO:0004386 : helicase activity<br>GO:0005515 : protein binding
CG4878	*eIF3-S9*	GO:0006413 : translational initiation	GO:0005829 : cytosol<br>GO:0005852 : eukaryotic translation initiation factor 3 complex	GO:0003729 : mRNA binding<br>GO:0000166 : nucleotide binding<br>GO:0003743 : translation initiation factor activity
CG4886	*cyp33*	GO:0000398 : nuclear mRNA splicing, via spliceosome<br>GO:0006911 : phagocytosis, engulfment<br>GO:0006457 : protein folding	GO:0071013 : catalytic step 2 spliceosome<br>GO:0005634 : nucleus<br>GO:0071011 : precatalytic spliceosome	GO:0003729 : mRNA binding<br>GO:0000166 : nucleotide binding<br>GO:0003755 : peptidyl-prolyl cis-trans isomerase activity

				GO:0003723 : RNA binding
CG4887		GO:0000398 : nuclear mRNA splicing, via spliceosome	GO:0071011 : precatalytic spliceosome	GO:0003729 : mRNA binding<br>GO:0003676 : nucleic acid binding<br>GO:0000166 : nucleotide binding<br>GO:0008270 : zinc ion binding
CG4912	*eEF1∂*	GO:0006414 : translational elongation	GO:0005853 : eukaryotic translation elongation factor 1 complex<br>GO:0005875 : microtubule associated complex	GO:0005085 : guanyl-nucleotide exchange factor activity<br>GO:0003746 : translation elongation factor activity
CG4954	*eIF3-S8*	GO:0006413 : translational initiation	GO:0005829 : cytosol<br>GO:0005852 : eukaryotic translation initiation factor 3 complex	GO:0003743 : translation initiation factor activity
CG5168				GO:0046872 : metal ion binding
CG5263	*smg*	GO:0051236 : establishment of RNA localization<br>GO:0017148 : negative regulation of translation<br>GO:0000289 : nuclear-transcribed mRNA poly(A) tail shortening<br>GO:0043488 : regulation of mRNA stability	GO:0005737 : cytoplasm<br>GO:0000932 : cytoplasmic mRNA processing body	GO:0003730 : mRNA 3'-UTR binding<br>GO:0003729 : mRNA binding<br>GO:0005515 : protein binding<br>GO:0030371 : translation repressor activity<br>GO:0000900 : translation repressor activity, nucleic acid binding
CG5439		GO:0007154 : cell communication		GO:0035091 : phosphatidylinositol binding
CG5589				GO:0005524 : ATP binding<br>GO:0004004 : ATP-dependent RNA helicase activity<br>GO:0003729 : mRNA binding
CG5605	*eRF1*	GO:0048102 : autophagic cell death<br>GO:0006605 : protein targeting<br>GO:0035071 : salivary gland cell autophagic cell death<br>GO:0007224 : smoothened signaling pathway<br>GO:0006415 : translational termination	GO:0005737 : cytoplasm<br>GO:0005829 : cytosol<br>GO:0018444 : translation release factor complex	GO:0003747 : translation release factor activity<br>GO:0016149 : translation release factor activity, codon specific<br>GO:0008079 : translation termination factor activity

CG5637	*nos*	GO:0008595 : anterior/posterior axis specification, embryo<br>GO:0001709 : cell fate determination<br>GO:0048813 : dendrite morphogenesis<br>GO:0016321 : female meiosis chromosome segregation<br>GO:0007281 : germ cell development<br>GO:0008354 : germ cell migration<br>GO:0048134 : germ-line cyst formation<br>GO:0042078 : germ-line stem cell division<br>GO:0030718 : germ-line stem cell maintenance<br>GO:0006378 : mRNA polyadenylation<br>GO:0043066 : negative regulation of apoptotic process<br>GO:0045892 : negative regulation of transcription, DNA-dependent<br>GO:0017148 : negative regulation of translation<br>GO:0007314 : oocyte anterior/posterior axis specification<br>GO:0048477 : oogenesis<br>GO:0007280 : pole cell migration<br>GO:0035282 : segmentation<br>GO:0007283 : spermatogenesis	GO:0045495 : pole plasm	GO:0005515 : protein binding<br>GO:0003723 : RNA binding<br>GO:0008270 : zinc ion binding
CG5705		GO:0006415 : translational termination	GO:0005739 : mitochondrion	GO:0016149 : translation release factor activity, codon specific
CG5800		GO:0007526 : larval somatic muscle development<br>GO:0022008 : neurogenesis		GO:0005524 : ATP binding<br>GO:0008026 : ATP-dependent helicase activity<br>GO:0003676 : nucleic acid binding<br>GO:0003724 : RNA helicase activity
CG6341	*Ef1ß*	GO:0006414 : translational elongation	GO:0005829 : cytosol<br>GO:0005853 : eukaryotic translation elongation factor 1 complex<br>GO:0005811 : lipid particle	GO:0005085 : guanyl-nucleotide exchange factor activity<br>GO:0003746 : translation elongation factor activity<br>GO:0003747 : translation release factor activity
CG6375	*pit*		GO:0005730 : nucleolus<br>GO:0005634 : nucleus	GO:0005524 : ATP binding<br>GO:0004004 : ATP-dependent RNA helicase activity

				GO:0003724 : RNA helicase activity<br>GO:0003676 : nucleic acid binding
CG6412	*mtEF-Ts*	GO:0006414 : translational elongation	GO:0005739 : mitochondrion	GO:0003746 : translation elongation factor activity
CG6418				GO:0005524 : ATP binding<br>GO:0004004 : ATP-dependent RNA helicase activity<br>GO:0004386 : helicase activity<br>GO:0003676 : nucleic acid binding
CG6493	*Dcr-2*	GO:0006342 : chromatin silencing<br>GO:0051607 : defense response to virus<br>GO:0009597 : detection of virus<br>GO:0033227 : dsRNA transport<br>GO:0070868 : heterochromatin organization involved in chromatin silencing<br>GO:0007626 : locomotory behavior<br>GO:0006379 : mRNA cleavage<br>GO:0031054 : pre-miRNA processing<br>GO:0030422 : production of siRNA involved in RNA interference<br>GO:0016246 : RNA interference<br>GO:0035087 : siRNA loading onto RISC involved in RNA interference<br>GO:0030423 : targeting of mRNA for destruction involved in RNA interference	GO:0005737 : cytoplasm<br>GO:0005634 : nucleus<br>GO:0016442 : RNA-induced silencing complex	GO:0005524 : ATP binding<br>GO:0008026 : ATP-dependent helicase activity<br>GO:0016887 : ATPase activity<br>GO:0003725 : double-stranded RNA binding<br>GO:0004386 : helicase activity<br>GO:0004525 : ribonuclease III activity<br>GO:0035197 : siRNA binding
CG6539	*Gem3*	GO:0007528 : neuromuscular junction development<br>GO:0022618 : ribonucleoprotein complex assembly	GO:0015030 : Cajal body<br>GO:0005737 : cytoplasm<br>GO:0071254 : cytoplasmic U snRNP body	GO:0005524 : ATP binding<br>GO:0004004 : ATP-dependent RNA helicase activity<br>GO:0003676 : nucleic acid binding<br>GO:0003724 : RNA helicase activity
CG6779	*RpS3*	GO:0006281 : DNA repair<br>GO:0007067 : mitosis	GO:0005737 : cytoplasm<br>GO:0022627 : cytosolic small ribosomal subunit	GO:0003906 : DNA-(apurinic or apyrimidinic site) lyase activity<br>GO:0008534 : oxidized purine base lesion DNA N-glycosylase activity

		GO:0043524 : negative regulation of neuron apoptotic process<br>GO:0006974 : response to DNA damage stimulus<br>GO:0006412 : translation	GO:0005811 : lipid particle<br>GO:0005875 : microtubule associated complex<br>GO:0016363 : nuclear matrix<br>GO:0005634 : nucleus<br>GO:0005840 : ribosome	GO:0003723 : RNA binding<br>GO:0003735 : structural constituent of ribosome
CG6866	*loqs*	GO:0007417 : central nervous system development<br>GO:0048132 : female germ-line stem cell division<br>GO:0030718 : germ-line stem cell maintenance<br>GO:0010586 : miRNA metabolic process<br>GO:0031054 : pre-miRNA processing<br>GO:0030422 : production of siRNA involved in RNA interference<br>GO:0070918 : production of small RNA involved in gene silencing by RNA<br>GO:0070920 : regulation of production of small RNA involved in gene silencing by RNA	GO:0005737 : cytoplasm	GO:0003725 : double-stranded RNA binding<br>GO:0005515 : protein binding
CG6946	*glo*	GO:0051276 : chromosome organization<br>GO:0060810 : intracellular mRNA localization involved in pattern specification process<br>GO:0007311 : maternal specification of dorsal/ventral axis, oocyte, germ-line encoded<br>GO:0048477 : oogenesis<br>GO:0045451 : pole plasm oskar mRNA localization<br>GO:0006417 : regulation of translation	GO:0043234 : protein complex	GO:0003729 : mRNA binding<br>GO:0003676 : nucleic acid binding<br>GO:0000166 : nucleotide binding<br>GO:0005515 : protein binding
CG6961				GO:0003729 : mRNA binding<br>GO:0003676 : nucleic acid binding<br>GO:0000166 : nucleotide binding
CG6987	*SF2*	GO:0007095 : mitotic cell cycle G2/M transition DNA damage checkpoint<br>GO:0006376 : mRNA splice site selection	GO:0071013 : catalytic step 2 spliceosome<br>GO:0005634 : nucleus	GO:0003729 : mRNA binding<br>GO:0003676 : nucleic acid binding

		GO:0022008 : neurogenesis<br>GO:0000398 : nuclear mRNA splicing, via spliceosome	GO:0071011 : precatalytic spliceosome<br>GO:0005681 : spliceosomal complex	GO:0000166 : nucleotide binding<br>GO:0005515 : protein binding
CG7082	*papi*			GO:0003723 : RNA binding
CG7269	*hel25E*	GO:0007052 : mitotic spindle organization<br>GO:0006406 : mRNA export from nucleus<br>GO:0000398 : nuclear mRNA splicing, via spliceosome<br>GO:0000381 : regulation of alternative nuclear mRNA splicing, via spliceosome	GO:0005875 : microtubule associated complex<br>GO:0005634 : nucleus<br>GO:0005681 : spliceosomal complex	GO:0005524 : ATP binding<br>GO:0004004 : ATP-dependent RNA helicase activity<br>GO:0003676 : nucleic acid binding<br>GO:0003724 : RNA helicase activity
CG7903				GO:0003729 : mRNA binding<br>GO:0003676 : nucleic acid binding<br>GO:0000166 : nucleotide binding
CG8053	*eIF-1a*	GO:0000022 : mitotic spindle elongation<br>GO:0007052 : mitotic spindle organization<br>GO:0006911 : phagocytosis, engulfment<br>GO:0007224 : smoothened signaling pathway<br>GO:0006412 : translation<br>GO:0006413 : translational initiation	GO:0005829 : cytosol<br>GO:0016282 : eukaryotic 43S preinitiation complex	GO:0003743 : translation initiation factor activity
CG8190	*eIF2B-gamma*	GO:0071456 : cellular response to hypoxia<br>GO:0006413 : translational initiation	GO:0005829 : cytosol<br>GO:0005851 : eukaryotic translation initiation factor 2B complex	GO:0016779 : nucleotidyltransferase activity<br>GO:0003743 : translation initiation factor activity
CG8205	*fus*	GO:0007173 : epidermal growth factor receptor signaling pathway		GO:0003729 : mRNA binding<br>GO:0003676 : nucleic acid binding<br>GO:0000166 : nucleotide binding
CG8280	*Ef1α48D*	GO:0008340 : determination of adult lifespan<br>GO:0006412 : translation<br>GO:0006414 : translational elongation	GO:0005737 : cytoplasm<br>GO:0005829 : cytosol<br>GO:0005853 : eukaryotic translation elongation factor 1 complex<br>GO:0005811 : lipid particle<br>GO:0005875 : microtubule associated complex	GO:0005525 : GTP binding<br>GO:0003924 : GTPase activity<br>GO:0003746 : translation elongation factor activity

CG8636	*eIF3-S4*	GO:0007067 : mitosis<br>GO:0000022 : mitotic spindle elongation<br>GO:0007052 : mitotic spindle organization<br>GO:0006413 : translational initiation	GO:0005852 : eukaryotic translation initiation factor 3 complex	GO:0003729 : mRNA binding<br>GO:0003676 : nucleic acid binding<br>GO:0000166 : nucleotide binding<br>GO:0003743 : translation initiation factor activity<br>GO:0008270 : zinc ion binding
CG8730	*drosha*	GO:0022008 : neurogenesis<br>GO:0031053 : primary miRNA processing<br>GO:0016075 : rRNA catabolic process	GO:0005634 : nucleus	GO:0003725 : double-stranded RNA binding<br>GO:0004525 : ribonuclease III activity
CG8882	*eIF3-S2 (Trip1)*	GO:0006412 : translation<br>GO:0006413 : translational initiation	GO:0005829 : cytosol<br>GO:0005852 : eukaryotic translation initiation factor 3 complex<br>GO:0005875 : microtubule associated complex	GO:0003743 : translation initiation factor activity
CG9054	*Ddx1*	GO:0001700 : embryonic development via the syncytial blastoderm<br>GO:0006446 : regulation of translational initiation<br>GO:0042254 : ribosome biogenesis<br>GO:0000245 : spliceosomal complex assembly	GO:0005575 : cellular_component	GO:0005524 : ATP binding<br>GO:0008026 : ATP-dependent helicase activity<br>GO:0004386 : helicase activity<br>GO:0003676 : nucleic acid binding
CG9075	*eIF-4a*	GO:0051297 : centrosome organization<br>GO:0006268 : DNA unwinding involved in replication<br>GO:0009950 : dorsal/ventral axis specification<br>GO:0048132 : female germ-line stem cell division<br>GO:0030718 : germ-line stem cell maintenance<br>GO:0007446 : imaginal disc growth<br>GO:0002168 : instar larval development<br>GO:0007067 : mitosis<br>GO:0000022 : mitotic spindle elongation<br>GO:0007052 : mitotic spindle organization<br>GO:0048477 : oogenesis	GO:0005813 : centrosome<br>GO:0005829 : cytosol<br>GO:0016281 : eukaryotic translation initiation factor 4F complex<br>GO:0005875 : microtubule associated complex<br>GO:0072686 : mitotic spindle<br>GO:0005634 : nucleus	GO:0005524 : ATP binding<br>GO:0008026 : ATP-dependent helicase activity<br>GO:0003729 : mRNA binding<br>GO:0000339 : RNA cap binding<br>GO:0003724 : RNA helicase activity<br>GO:0017116 : single-stranded DNA-dependent ATP-dependent DNA helicase activity<br>GO:0003743 : translation initiation factor activity

		GO:0000381 : regulation of alternative nuclear mRNA splicing, via spliceosome<br>GO:0006974 : response to DNA damage stimulus<br>GO:0006413 : translational initiation		
CG9107				GO:0000166 : nucleotide binding
CG9124	*eIF-3p40*	GO:0022008 : neurogenesis<br>GO:0006413 : translational initiation	GO:0005829 : cytosol<br>GO:0005852 : eukaryotic translation initiation factor 3 complex	GO:0003743 : translation initiation factor activity
CG9218	*sm*	GO:0008343 : adult feeding behavior<br>GO:0007411 : axon guidance	GO:0030529 : ribonucleoprotein complex	GO:0003729 : mRNA binding<br>GO:0003676 : nucleic acid binding<br>GO:0000166 : nucleotide binding
CG9373	*rump*	GO:0008595 : anterior/posterior axis specification, embryo<br>GO:0009952 : anterior/posterior pattern specification<br>GO:0008298 : intracellular mRNA localization<br>GO:0007067 : mitosis<br>GO:0007277 : pole cell development<br>GO:0045451 : pole plasm *oskar* mRNA localization<br>GO:0035282 : segmentation	GO:0005737 : cytoplasm<br>GO:0005634 : nucleus<br>GO:0030529 : ribonucleoprotein complex	GO:0003730 : mRNA 3'-UTR binding<br>GO:0003729 : mRNA binding<br>GO:0000166 : nucleotide binding
CG9412	*rin*	GO:0001752 : compound eye photoreceptor fate commitment<br>GO:0000398 : nuclear mRNA splicing, via spliceosome<br>GO:0016318 : ommatidial rotation<br>GO:0007265 : Ras protein signal transduction<br>GO:0006810 : transport	GO:0071013 : catalytic step 2 spliceosome<br>GO:0005829 : cytosol<br>GO:0005811 : lipid particle<br>GO:0071011 : precatalytic spliceosome	GO:0003729 : mRNA binding<br>GO:0003676 : nucleic acid binding<br>GO:0000166 : nucleotide binding<br>GO:0017124 : SH3 domain binding
CG9680	*Dbp73D*	GO:0022008 : neurogenesis		GO:0005524 : ATP binding<br>GO:0008026 : ATP-dependent helicase activity<br>GO:0004004 : ATP-dependent RNA helicase activity
CG9755	*pum*	GO:0008595 : anterior/posterior axis specification, embryo<br>GO:0001709 : cell fate determination	GO:0005737 : cytoplasm	GO:0003730 : mRNA 3'-UTR binding<br>GO:0003723 : RNA binding

		GO:0016477 : cell migration<br>GO:0048813 : dendrite morphogenesis<br>GO:0007281 : germ cell development<br>GO:0042078 : germ-line stem cell division<br>GO:0008258 : head involution<br>GO:0007616 : long-term memory<br>GO:0007067 : mitosis<br>GO:0045786 : negative regulation of cell cycle<br>GO:0042059 : negative regulation of epidermal growth factor receptor signaling pathway<br>GO:0045892 : negative regulation of transcription, DNA-dependent<br>GO:0017148 : negative regulation of translation<br>GO:0000288 : nuclear-transcribed mRNA catabolic process, deadenylation-dependent decay<br>GO:0048477 : oogenesis<br>GO:0007280 : pole cell migration<br>GO:0045727 : positive regulation of translation<br>GO:0008582 : regulation of synaptic growth at neuromuscular junction<br>GO:0050804 : regulation of synaptic transmission<br>GO:0007268 : synaptic transmission		GO:0000900 : translation repressor activity, nucleic acid binding
CG9769	*eIF3-S5*	GO:0048102 : autophagic cell death<br>GO:0035071 : salivary gland cell autophagic cell death<br>GO:0006413 : translational initiation	GO:0005852 : eukaryotic translation initiation factor 3 complex	GO:0003743 : translation initiation factor activity
CG9809	*Spargel*	GO:0008286 : insulin receptor signaling pathway<br>GO:0046627 : negative regulation of insulin receptor signaling pathway<br>GO:0010821 : regulation of mitochondrion organization		GO:0003729 : mRNA binding<br>GO:0003676 : nucleic acid binding<br>GO:0000166 : nucleotide binding

		GO:0006355 : regulation of transcription, DNA-dependent		
CG9841	*Efsec*	GO:0006414 : translational elongation<br>GO:0006451 : translational readthrough		GO:0005525 : GTP binding<br>GO:0003924 : GTPase activity<br>GO:0003746 : translation elongation factor activity
CG9946	*eIF-2α*	GO:0001677 : formation of translation initiation ternary complex<br>GO:0000022 : mitotic spindle elongation<br>GO:0007052 : mitotic spindle organization<br>GO:0006413 : translational initiation	GO:0005829 : cytosol<br>GO:0005850 : eukaryotic translation initiation factor 2 complex<br>GO:0005851 : eukaryotic translation initiation factor 2B complex<br>GO:0005875 : microtubule associated complex	GO:0005525 : GTP binding<br>GO:0003743 : translation initiation factor activity<br>GO:0000049 : tRNA binding
CG10084	*swm*	GO:0006397 : mRNA processing<br>GO:0045879 : negative regulation of smoothened signaling pathway<br>GO:0007346 : regulation of mitotic cell cycle	GO:0005634 : nucleus	GO:0003676 : nucleic acid binding<br>GO:0000166 : nucleotide binding<br>GO:0008270 : zinc ion binding
CG10124	*eIF4E-4*	GO:0006413 : translational initiation	GO:0016281 : eukaryotic translation initiation factor 4F complex	GO:0031370 : eukaryotic initiation factor 4G binding<br>GO:0005515 : protein binding<br>GO:0000340 : RNA 7-methylguanosine cap binding<br>GO:0000339 : RNA cap binding<br>GO:0003743 : translation initiation factor activity
CG10203	*x16*	GO:0006376 : mRNA splice site selection<br>GO:0007399 : nervous system development<br>GO:0000398 : nuclear mRNA splicing, via spliceosome<br>GO:0008380 : RNA splicing	GO:0071013 : catalytic step 2 spliceosome<br>GO:0005634 : nucleus<br>GO:0071011 : precatalytic spliceosome<br>GO:0005681 : spliceosomal complex	GO:0003729 : mRNA binding<br>GO:0003676 : nucleic acid binding<br>GO:0000166 : nucleotide binding<br>GO:0005515 : protein binding<br>GO:0003723 : RNA binding<br>GO:0008270 : zinc ion binding
CG10315	*eIF2B-∂*	GO:0022008 : neurogenesis<br>GO:0006413 : translational initiation	GO:0005829 : cytosol<br>GO:0005851 : eukaryotic translation initiation factor 2B complex	GO:0003743 : translation initiation factor activity
CG10466		GO:0000398 : nuclear mRNA splicing, via spliceosome	GO:0071013 : catalytic step 2 spliceosome	GO:0003729 : mRNA binding

			GO:0071011 : precatalytic spliceosome	GO:0003676 : nucleic acid binding<br>GO:0000166 : nucleotide binding
CG10719	*brat*	GO:0008356 : asymmetric cell division<br>GO:0055060 : asymmetric neuroblast division resulting in ganglion mother cell formation<br>GO:0007411 : axon guidance<br>GO:0007420 : brain development<br>GO:0007402 : ganglion mother cell fate determination<br>GO:0008285 : negative regulation of cell proliferation<br>GO:0007406 : negative regulation of neuroblast proliferation<br>GO:0007405 : neuroblast proliferation<br>GO:0022008 : neurogenesis<br>GO:0030182 : neuron differentiation<br>GO:0048477 : oogenesis<br>GO:0006911 : phagocytosis, engulfment<br>GO:0051726 : regulation of cell cycle<br>GO:0006417 : regulation of translation<br>GO:0016072 : rRNA metabolic process<br>GO:0009303 : rRNA transcription<br>GO:0035282 : segmentation	GO:0035282 : segmentation<br>GO:0045180 : basal cortex<br>GO:0005737 : cytoplasm	GO:0005515 : protein binding<br>GO:0045182 : translation regulator activity<br>GO:0030371 : translation repressor activity<br>GO:0008270 : zinc ion binding
CG10777				GO:0005524 : ATP binding<br>GO:0008026 : ATP-dependent helicase activity<br>GO:0003676 : nucleic acid binding<br>GO:0003724 : RNA helicase activity
CG10868	*orb*	GO:0000578 : embryonic axis specification<br>GO:0007281 : germ cell development<br>GO:0048134 : germ-line cyst formation<br>GO:0007294 : germarium-derived oocyte fate determination<br>GO:0008298 : intracellular mRNA localization<br>GO:0006378 : mRNA polyadenylation<br>GO:0007314 : oocyte anterior/posterior axis specification	GO:0005737 : cytoplasm	GO:0003729 : mRNA binding<br>GO:0000166 : nucleotide binding<br>GO:0005515 : protein binding<br>GO:0017131 : uridine-rich cytoplasmic polyadenylylation element binding

		GO:0007309 : oocyte axis specification<br>GO:0007310 : oocyte dorsal/ventral axis specification<br>GO:0048477 : oogenesis<br>GO:0007315 : pole plasm assembly<br>GO:0045451 : pole plasm oskar mRNA localization<br>GO:0046012 : positive regulation of oskar mRNA translation<br>GO:0045727 : positive regulation of translation<br>GO:0046011 : regulation of oskar mRNA translation		
CG10881	*eIF3-S4*	GO:0006413 : translational initiation	GO:0005852 : eukaryotic translation initiation factor 3 complex	GO:0003729 : mRNA binding<br>GO:0003676 : nucleic acid binding<br>GO:0000166 : nucleotide binding<br>GO:0003743 : translation initiation factor activity<br>GO:0008270 : zinc ion binding
CG10901	*osk*	GO:0008595 : anterior/posterior axis specification, embryo<br>GO:0007281 : germ cell development<br>GO:0007616 : long-term memory<br>GO:0046594 : maintenance of pole plasm mRNA location<br>GO:0007314 : oocyte anterior/posterior axis specification<br>GO:0008103 : oocyte microtubule cytoskeleton polarization<br>GO:0048477 : oogenesis<br>GO:0030719 : P granule organization<br>GO:0007277 : pole cell development<br>GO:0007278 : pole cell fate determination<br>GO:0007279 : pole cell formation<br>GO:0007315 : pole plasm assembly<br>GO:0019094 : pole plasm mRNA localization<br>GO:0007318 : pole plasm protein localization<br>GO:0045727 : positive regulation of translation	GO:0043186 : P granule<br>GO:0045495 : pole plasm	GO:0016787 : hydrolase activity

		GO:0007359 : posterior abdomen determination<br>GO:0043488 : regulation of mRNA stability<br>GO:0046011 : regulation of oskar mRNA translation<br>GO:0007632 : visual behavior<br>GO:0008542 : visual learning		
CG11266		GO:0000398 : nuclear mRNA splicing, via spliceosome<br>GO:0000381 : regulation of alternative nuclear mRNA splicing, via spliceosome<br>GO:0008380 : RNA splicing	GO:0071013 : catalytic step 2 spliceosome<br>GO:0005634 : nucleus<br>GO:0071011 : precatalytic spliceosome<br>GO:0005681 : spliceosomal complex	GO:0003729 : mRNA binding<br>GO:0000166 : nucleotide binding<br>GO:0003723 : RNA binding
CG11334		GO:0006412 : translation<br>GO:0035220 : wing disc development	GO:0005851 : eukaryotic translation initiation factor 2B complex	GO:0045182 : translation regulator activity
CG11454				GO:0003729 : mRNA binding<br>GO:0003676 : nucleic acid binding<br>GO:0000166 : nucleotide binding
CG11505		GO:0008150 : biological_process	GO:0005575 : cellular_component	GO:0003676 : nucleic acid binding
CG11726				GO:0003729 : mRNA binding<br>GO:0003676 : nucleic acid binding<br>GO:0000166 : nucleotide binding
CG12493			GO:0005634 : nucleus	GO:0003725 : double-stranded RNA binding<br>GO:0035197 : siRNA binding
CG13425	*bl*	GO:0035107 : appendage morphogenesis<br>GO:0045165 : cell fate commitment<br>GO:0008283 : cell proliferation<br>GO:0007446 : imaginal disc growth<br>GO:0000381 : regulation of alternative nuclear mRNA splicing, via spliceosome	GO:0000785 : chromatin<br>GO:0005737 : cytoplasm<br>GO:0005634 : nucleus<br>GO:0035062 : omega speckle<br>GO:0030529 : ribonucleoprotein complex	GO:0003729 : mRNA binding<br>GO:0003723 : RNA binding<br>GO:0008134 : transcription factor binding
CG14718			GO:0005622 : intracellular	GO:0003729 : mRNA binding<br>GO:0003676 : nucleic acid binding<br>GO:0000166 : nucleotide binding<br>GO:0008270 : zinc ion binding
CG14891				GO:0003676 : nucleic acid binding<br>GO:0000166 : nucleotide binding

CG16901	*sqd*	GO:0008069 : dorsal/ventral axis specification, ovarian follicular epithelium<br>GO:0009953 : dorsal/ventral pattern formation<br>GO:0007293 : germarium-derived egg chamber formation<br>GO:0008298 : intracellular mRNA localization<br>GO:0006406 : mRNA export from nucleus<br>GO:0033119 : negative regulation of RNA splicing<br>GO:0017148 : negative regulation of translation<br>GO:0000398 : nuclear mRNA splicing, via spliceosome<br>GO:0000184 : nuclear-transcribed mRNA catabolic process, nonsense-mediated decay<br>GO:0007314 : oocyte anterior/posterior axis specification<br>GO:0030720 : oocyte localization involved in germarium-derived egg chamber formation<br>GO:0016325 : oocyte microtubule cytoskeleton organization<br>GO:0048477 : oogenesis<br>GO:0007297 : ovarian follicle cell migration<br>GO:0019094 : pole plasm mRNA localization<br>GO:0045451 : pole plasm oskar mRNA localization<br>GO:0000381 : regulation of alternative nuclear mRNA splicing, via spliceosome<br>GO:0006405 : RNA export from nucleus	GO:0071013 : catalytic step 2 spliceosome<br>GO:0000785 : chromatin<br>GO:0005737 : cytoplasm<br>GO:0005730 : nucleolus<br>GO:0005634 : nucleus<br>GO:0035062 : omega speckle<br>GO:0005703 : polytene chromosome puff<br>GO:0071011 : precatalytic spliceosome<br>GO:0030529 : ribonucleoprotein complex	GO:0003730 : mRNA 3'-UTR binding<br>GO:0003729 : mRNA binding<br>GO:0003676 : nucleic acid binding<br>GO:0000166 : nucleotide binding
CG17492	*mib2*	GO:0046716 : muscle cell homeostasis<br>GO:0007520 : myoblast fusion<br>GO:0016567 : protein ubiquitination	GO:0005737 : cytoplasm<br>GO:0031430 : M band<br>GO:0030018 : Z disc	GO:0032038 : myosin II heavy chain binding<br>GO:0032033 : myosin II light chain binding<br>GO:0004842 : ubiquitin-protein ligase activity<br>GO:0008270 : zinc ion binding
CG17686	*DIP1*		GO:0000785 : chromatin	GO:0003682 : chromatin binding

			GO:0005737 : cytoplasm<br>GO:0005634 : nucleus	GO:0003725 : double-stranded RNA binding<br>GO:0070883 : pre-miRNA binding<br>GO:0005515 : protein binding
CG18259				GO:0003676 : nucleic acid binding<br>GO:0000166 : nucleotide binding
CG31061	*Gr98d*	GO:0050909 : sensory perception of taste	GO:0016021 : integral to membrane	GO:0008527 : taste receptor activity
CG31762	*aret (bruno)*	GO:0007281 : germ cell development<br>GO:0042078 : germ-line stem cell division<br>GO:0030727 : germarium-derived female germ-line cyst formation<br>GO:0002121 : inter-male aggressive behavior<br>GO:0006378 : mRNA polyadenylation<br>GO:0007319 : negative regulation of oskar mRNA translation<br>GO:0017148 : negative regulation of translation<br>GO:0048477 : oogenesis<br>GO:0031536 : positive regulation of exit from mitosis<br>GO:0000381 : regulation of alternative nuclear mRNA splicing, via spliceosome<br>GO:0046011 : regulation of oskar mRNA translation<br>GO:0007286 : spermatid development	GO:0005634 : nucleus<br>GO:0043186 : P granule	GO:0003730 : mRNA 3'-UTR binding<br>GO:0003729 : mRNA binding<br>GO:0000166 : nucleotide binding<br>GO:0005515 : protein binding<br>GO:0003723 : RNA binding
CG32423	*shep*	GO:0042332 : gravitaxis	GO:0005575 : cellular_component	GO:0003729 : mRNA binding<br>GO:0000166 : nucleotide binding<br>GO:0003723 : RNA binding
CG32706		GO:0008150 : biological_process	GO:0005575 : cellular_component	GO:0003676 : nucleic acid binding<br>GO:0000166 : nucleotide binding
CG33100	*4EHP*	GO:0002121 : inter-male aggressive behavior<br>GO:0017148 : negative regulation of translation<br>GO:0022008 : neurogenesis<br>GO:0006413 : translational initiation	GO:0016281 : eukaryotic translation initiation factor 4F complex	GO:0031370 : eukaryotic initiation factor 4G binding<br>GO:0005515 : protein binding<br>GO:0000340 : RNA 7-methylguanosine cap binding<br>GO:0003743 : translation initiation factor activity<br>GO:0030371 : translation repressor activity

CG33106	*mask*	GO:0019730 : antimicrobial humoral response<br>GO:0008283 : cell proliferation<br>GO:0009987 : cellular process<br>GO:0051298 : centrosome duplication<br>GO:0001751 : compound eye photoreceptor cell differentiation<br>GO:0007016 : cytoskeletal anchoring at plasma membrane<br>GO:0007169 : transmembrane receptor protein tyrosine kinase signaling pathway	GO:0005811 : lipid particle	GO:0003723 : RNA binding<br>GO:0005200 : structural constituent of cytoskeleton
CG33197	*mbl*	GO:0006915 : apoptotic process<br>GO:0001751 : compound eye photoreceptor cell differentiation<br>GO:0009790 : embryo development<br>GO:0007517 : muscle organ development<br>GO:0007422 : peripheral nervous system development<br>GO:0045924 : regulation of female receptivity<br>GO:0007525 : somatic muscle development	GO:0005737 : cytoplasm<br>GO:0005634 : nucleus<br>GO:0048471 : perinuclear region of cytoplasm	GO:0003677 : DNA binding<br>GO:0003676 : nucleic acid binding<br>GO:0008270 : zinc ion binding
CG34354				GO:0003729 : mRNA binding<br>GO:0003676 : nucleic acid binding<br>GO:0000166 : nucleotide binding
CG40351	*Set1*	GO:0006338 : chromatin remodeling<br>GO:0051568 : histone H3-K4 methylation<br>GO:0080182 : histone H3-K4 trimethylation	GO:0005634 : nucleus<br>GO:0048188 : Set1C/COMPASS complex	GO:0042800 : histone methyltransferase activity (H3-K4 specific)<br>GO:0018024 : histone-lysine N-methyltransferase activity<br>GO:0003676 : nucleic acid binding<br>GO:0000166 : nucleotide binding
